# Multimodal cue integration and learning in a neural representation of head direction

**DOI:** 10.1038/s41593-024-01823-z

**Published:** 2025-07-23

**Authors:** Melanie A. Basnak, Anna Kutschireiter, Tatsuo S. Okubo, Albert Chen, Pavel Gorelik, Jan Drugowitsch, Rachel I. Wilson

**Affiliations:** Department of Neurobiology, Harvard Medical School, Boston, MA USA

**Keywords:** Spatial memory, Network models

## Abstract

Navigation requires us to take account of multiple spatial cues with varying levels of informativeness and learn their spatial relationships. Here we investigate this process in the *Drosophila* head direction system, which functions as a ring attractor and a topographic map of head direction. Using population calcium imaging and multimodal virtual reality environments, we show that increasing cue informativeness improves encoding accuracy and produces a narrower and higher bump of activity. When cues conflict, the more informative cue exerts more weight. A familiar cue is weighted more heavily and used to guide the remapping of a less familiar cue. When a cue is less informative, it is remapped more readily in response to cue conflict. All these results can be explained by an attractor model with plastic sensory synapses. Our findings provide a mechanistic explanation for how the brain assembles spatial representations through inference and learning.

## Abstract (web summary)

Basnak et al. show that particularly informative spatial cues produce a higher and narrower bump of activity in the *Drosophila* head direction system. More informative cues can instruct the remapping of other cues to produce a self-consistent map.

## Main

When we enter a new environment, we encounter a host of cues that might be useful for guiding navigation. Some cues are more useful than others because they are more salient or easily located; for example, the moon is more visible than a star. At the same time, the usefulness of a spatial cue also depends on its stability^1^. For example, a strong wind is salient because it can easily inform us about the direction we are facing, but only if the wind is blowing from a stable direction. Similarly, a faraway mountain and a nearby tree may be equally salient as visual objects but only the mountain will have a stable position on the horizon and, thus, it is much more informative about the direction we are facing. For this reason, we should assess the stability of each external cue by monitoring its position over time, relative to our own self-motion cues. As we acquire more familiarity with an external cue, we should logically ascribe it more weight (relative to self-motion cues), as long as the external cue appears stable^1,2^. Thus, a cue must be both stable and familiar to be highly informative for navigation.

Not surprisingly, behavioral studies have shown that navigating animals generally ascribe more weight to external cues that appear to be more stable and familiar, just as they ascribe more weight to cues that are more salient^3–14^. Moreover, neurophysiological studies in rats and mice have shown that salient or stable and familiar cues exert the strongest influence over the neurons in the brain’s navigation centers^15–17^. How does this work mechanistically? The brain’s navigation centers are thought to be organized around attractor networks (that is, networks with multiple stable states)^18^. Self-motion cues drive transitions between different stable states, creating a working memory of the organism’s position on a map or its orientation in space. Specifically, the attractors that correspond to the sense of direction are thought to be ring attractors, meaning that their stable states form a closed circle in network state space^19,20^. Self-movement signals during head rotations would push the network state around this circle. It has been proposed that the sensory weights onto these ring attractors might be dynamically adjusted according to a Hebbian learning rule^20–28^. This would automatically allocate more weight (stronger synaptic connections) to environmental cues that are more salient, stable and familiar. Once stable environmental cues are familiar and, thus, well learned, they should improve the ring attractor network’s ability to accurately track head direction (HD) beyond what it can achieve on the basis of self-movement signals alone^19,20^. Although this model was originally proposed to explain HD cells, it has also been extended to explain grid cells^29–31^. However, all of these models have been largely untested at a mechanistic level.

**Fig. 1:**
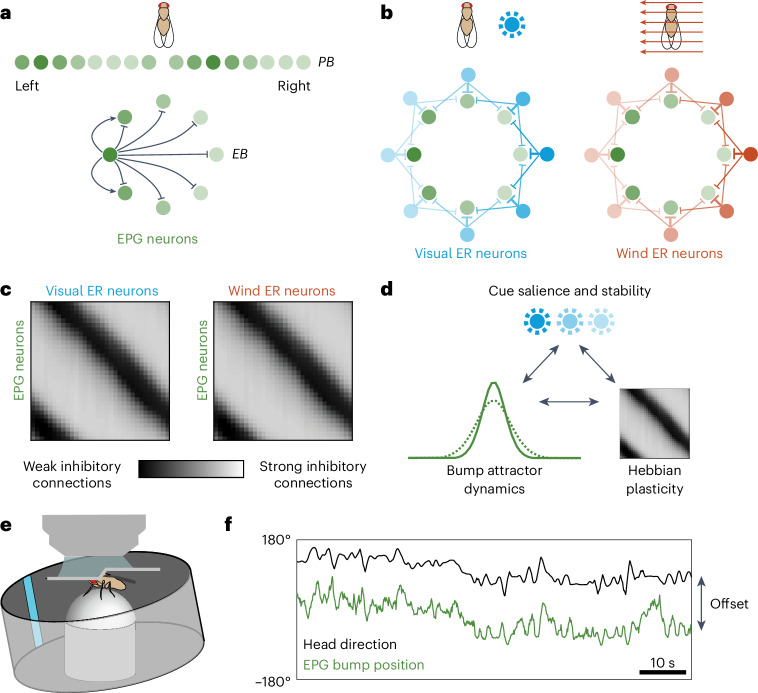
The *Drosophila* HD system. **a**, HD cells (EPG neurons) form a ring attractor network in the EB. Their axons project to the PB, where they form two linearized topographic maps of HD. **b**, The position of the EPG activity bump is influenced by ER neurons that encode the positions of visual HD cues or the direction of the wind. ER neurons are inhibitory and the most active ER neurons push the bump to the location where their inhibitory output is minimal. ER → EPG connections are anatomically all-to-all but their weights are shaped by Hebbian plasticity at ER → EPG synapses, such that each ER neuron generally makes functional synapses onto only a subset of EPG neurons. **c**, Schematic ER → EPG weights. Given a single visual cue and a steady wind direction, associative LTD is predicted to produce a diagonal notch of weak connections in each weight matrix. EPG neurons are sorted by their preferred HD. ER neurons are sorted by their preferred cue position. If the two cues are aligned in the simulated environment, Hebbian plasticity should align the notches. **d**, We hypothesize that cue salience and stability affect bump attractor dynamics and learning. **e**, We image EPG neurons in head-fixed flies walking on a spherical treadmill. As the fly turns on the spherical treadmill, the virtual environment rotates around the fly in the expected direction. Here, the environment contains a bright vertical stripe that serves as an HD cue. **f**, The bump of EPG activity tracks the fly’s fictive HD in a virtual reality environment, with a relatively constant angular offset. Bump position rotates clockwise in the EB (imaged from the posterior side of the head) as HD rotates counterclockwise; therefore, to account for this directionality, we always plot (−HD) to make it easier to visualize the correspondence between bump position and HD.

Recently, the *Drosophila* HD network has emerged as a useful system for testing these ideas. Genetic experiments have demonstrated that *Drosophila* HD cells are essential for navigation behavior^32,33^. All the HD cells in this network can be imaged simultaneously as a head-fixed fly navigates in a virtual reality environment^33,34^. Moreover, this network’s anatomical connectivity is known in detail from connectome data^35^. This network functions as a ring attractor, which is also a topographic map^36^ (a ‘bump attractor’); it exhibits a persistent bump of activity, whose position stores a working memory of the fly’s current orientation (Fig. 1a). The bump’s position changes smoothly as the fly rotates, reflecting the influence of self-motion cues^37,38^ and external sensory inputs^34,39^ (Fig. 1b). The pattern of connection weights from sensory cells onto HD cells can change during spatial learning according to a Hebbian learning rule, allowing this system to learn the pattern of visual cues in the current environment^22–24^ (Fig. 1c).

Here, we ask how the function of this network depends on cue salience and familiarity (Fig. 1d). We find that cue salience and familiarity alter the width and amplitude of the bump while also driving rapid spatial learning that continuously updates the properties of the network, such that salient and familiar cues are accorded more weight. Mechanistically, our results can be explained by a ring attractor model with a high rate of synaptic modification at sensory synapses onto HD cells. Conceptually, our findings show how continuous synaptic plasticity allows ongoing spatial learning and inference in a dynamic environment, albeit at the cost of reducing the stability of the system’s representational coordinate frame. Thus, our results highlight the fundamental tradeoff between stability and flexibility in the brain’s navigational centers.

## Results

To study cue integration and learning in the *Drosophila* HD system, we expressed jGCaMP7f (ref. ^40^) in HD cells (equivalence potential gradient (EPG) neurons) under Gal4–UAS control^41^ and we imaged the EPG ensemble using a two-photon microscope as the fly walked freely on a spherical treadmill (Fig. 1e). By measuring the rotational velocity of the sphere, we can infer the fly’s intended rotational velocity. If we then rotate a direction cue around the fly in closed loop with the ball’s rotation, the EPG ensemble can track the fly’s fictive HD (Fig. 1f). Note that the head and body are rigidly coupled in our experiments; thus, HD is always equal to heading. The fly rotates its fictive HD and heading by maneuvering on the spherical treadmill.

### Increasing cue intensity changes the bump profile

**Fig. 2:**
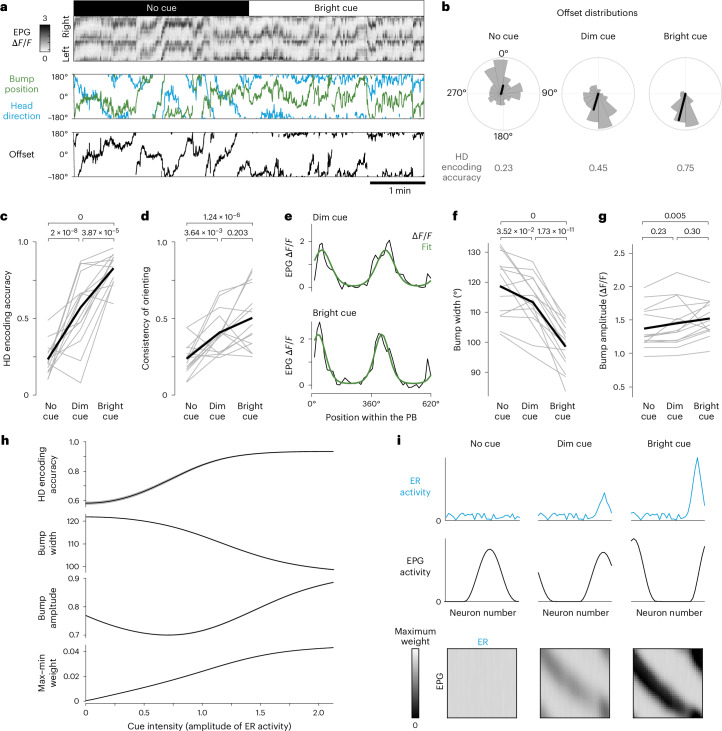
Increasing cue intensity changes the bump profile. This figure shows data for 15 flies. **a**, EPG *ΔF*/*F* in the PB is used to infer the position of the EPG bump in the EB (green). When a visual cue is present, the bump tracks HD (blue). The offset between HD and bump is fairly constant when a bright visual HD cue is available. **b**, Offset distributions for the fly in **a**. Black lines are vector averages, with line length denoting HD encoding accuracy. Polar histograms are shown on the same scale. **c**, HD encoding accuracy in each condition. In **c**,**d**,**f**,**g**, single flies are shown in gray, mean values are shown in black and *P* values are shown (linear mixed-effects models with Tukey comparisons and Bonferroni corrections). **d**, Consistency of behavioral orienting in each condition. **e**, *ΔF*/*F* and fit (Extended Data Fig. 1) at two time points for the same fly. **f**, Bump width in each condition. **g**, Bump amplitude in each condition. **h**, Model of the effect of increasing cue intensity on HD encoding accuracy, EPG bump width, EPG bump amplitude and the range of ER → EPG weights (max–min). Mean of 100 simulation runs ± s.e.m. **i**, Model of the spatial profile of ER and EPG population activity at one HD for each condition. Bottom, ER → EPG weights at the end of each block. The maximum weight represents maximum inhibition. The bright cue produces a deeper notch in the weight matrix.

First, we examined how cue salience affects bump attractor dynamics. In these experiments, we used intensity as a proxy for salience; we switched among a bright cue, a dim cue and no cue in randomized interleaved 200-s blocks. For each cue intensity, we measured the accuracy of HD encoding (Fig. 2a). A perfectly accurate HD system should have a constant offset between HD and bump position. We, therefore, measured the circular variance in the offset over the duration of each block and we defined ‘HD encoding accuracy’ as 1 − circular variance (Fig. 2b). We cannot directly measure the informativeness of a spatial cue by measuring its physical properties^14^ but we can use HD encoding accuracy as an operational measure of a cue’s informativeness.

In pilot experiments, we lowered the intensity of the dim cue until HD encoding accuracy was only slightly better than no cue at all. We then systematically compared HD encoding accuracy for all three cue conditions and we confirmed that HD encoding accuracy progressively improves with higher cue intensity (Fig. 2c); this demonstrates that the bright cue actually conveys more information. This makes sense because a bright cue should have a higher signal-to-noise ratio. Moreover, with higher cue intensity, we found that flies also tended to orient in a more consistent direction in virtual space (Fig. 2d). If the brain’s internal estimate of HD is more accurate, it is logical that this should enable a more consistent behavioral orientation because flies use their HD system to orient toward an internal goal direction^32,33,42^.

Notably, when we increased cue intensity, we also found changes in the bump profile. Specifically, bump width decreased in a graded manner as visual cue intensity increased (Fig. 2e,f), mirroring the graded increase in HD encoding accuracy. Meanwhile, bump amplitude increased (Fig. 2g), although this effect was only clear for the highest cue intensity.

To better understand why cue intensity should affect the bump profile, we modeled this network as a ring attractor with plastic sensory inputs. In this model, each EPG neuron excites its neighbors while also driving global inhibition^35,36^ (Fig. 1a). EPG neurons receive inhibitory input from ER neurons^23^, whose receptive fields tile visual space^43^ (Fig. 1b). In agreement with anatomical data^35^, ER → EPG connections are all-to-all, with weights governed by a Hebbian learning rule^22–24^ that weakens inhibition between coactive ER–EPG pairs through associative long-term depression (LTD). At the same time, this learning rule also strengthens inhibition onto EPG neurons that are active without ER input through nonassociative long-term potentiation (LTP), which depends on postsynaptic activity alone. EPG neurons also receive noisy self-motion signals that tend to push the EPG bump in the correct direction during turning maneuvers^37,38^. For each simulation iteration, we generated a random sequence of turning maneuvers that specify HD, ER input and self-motion input.

In this model, increasing the intensity of a visual cue increases the accuracy of HD encoding (Fig. 2h). It also decreases bump width (Fig. 2h). This is because ER neurons are inhibitory and increasing the intensity of a visual cue recruits more inhibitory drive to the network. Meanwhile, in this model, increasing visual cue intensity produces nonmonotonic changes in bump amplitude, reflecting two competing effects. On the one hand, increasing inhibitory drive pushes bump amplitude down. On the other hand, increasing presynaptic activity promotes associative LTD, producing a deeper notch in the pattern of ER → EPG weights (Fig. 2i), leading to stronger disinhibition of the most active EPG neurons; this pushes bump amplitude up. If the cue is very bright, the latter effect wins, producing a net increase in bump amplitude (Fig. 2h). In essence, the pattern of ER → EPG weights resembles the negative image of the cue and the intensity of that image reflects the intensity of the cue.

To summarize, we find that increases in cue brightness increase HD encoding accuracy and narrow the bump. Very bright cues also increase bump amplitude. All these changes in bump profile can be explained by the interaction of inhibitory sensory input and a Hebbian learning rule.

**Fig. 3:**
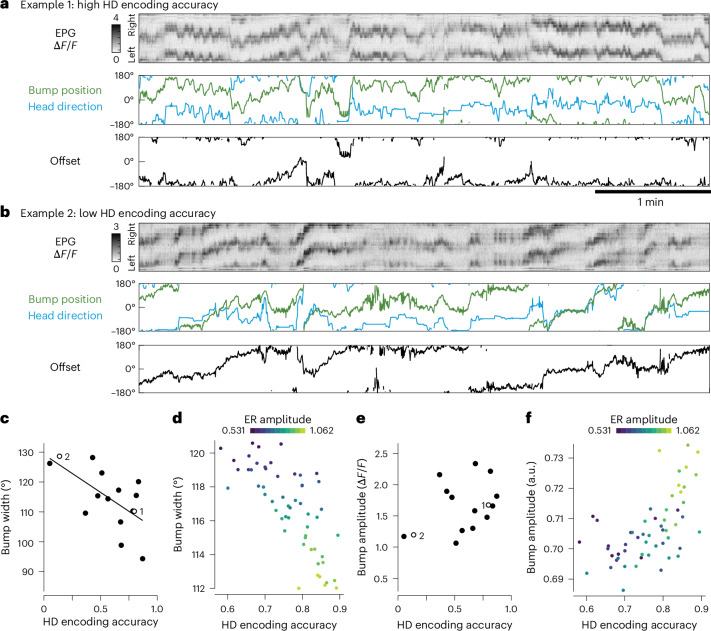
The influence of a cue varies across individuals. This figure shows data for the 15 flies from Fig. 2. **a**, An example fly navigating in the virtual environment with the dim visual cue (HD encoding accuracy = 0.80). The polar histogram shows offset distribution. **b**, Another fly in the same environment (HD encoding accuracy = 0.14). **c**, Individual variations in HD encoding accuracy correlate with bump width (*P* = 0.01, Pearson correlation). Data are from the first block where the dim visual cue was presented (200 s in duration). The dim cue produced the most individual variation (Extended Data Fig. 2). **d**, Model showing that, with the dim cue, small variations in ER amplitude produce strongly correlated changes in bump width and HD encoding accuracy. **e**, Individual variations in HD encoding accuracy do not correlate with bump amplitude (*P* = 0.18, Pearson correlation). All data are from the environment with the dim cue. **f**, Model showing that, with a dim cue, small variations in ER amplitude produce weakly correlated changes in bump amplitude and HD encoding accuracy.

Thus far, we focused on average trends across flies but it is also instructive to examine individual differences in bump dynamics. In particular, we noticed that the same virtual reality environment could produce high HD encoding accuracy in some individuals (Fig. 3a) but lower accuracy in other individuals (Fig. 3b). Moreover, these variations were correlated with individual differences in bump width (Fig. 3c). We can recapitulate these results in the model by generating individual variations in the overall level of visually evoked activity in ER neurons. This produces variations in HD encoding accuracy and correlated changes in bump width (Fig. 3d). Bump amplitude variations are not so well correlated with HD encoding accuracy, both in our data (Fig. 3e) and in the model (Fig. 3f). In the model, this is because an increase in ER activity can produce opposing effects on bump amplitude (Fig. 2h).

In short, our results show that different individuals can experience the same cue as conveying more or less information. This produces correlated variations across individuals in bump width and HD encoding accuracy. Our model suggests that these individual variations arise with differences in the intensity of sensory input to the HD system, which might arise from individual differences in how flies process the visual cue.

### More informative cues are accorded more weight

**Fig. 4:**
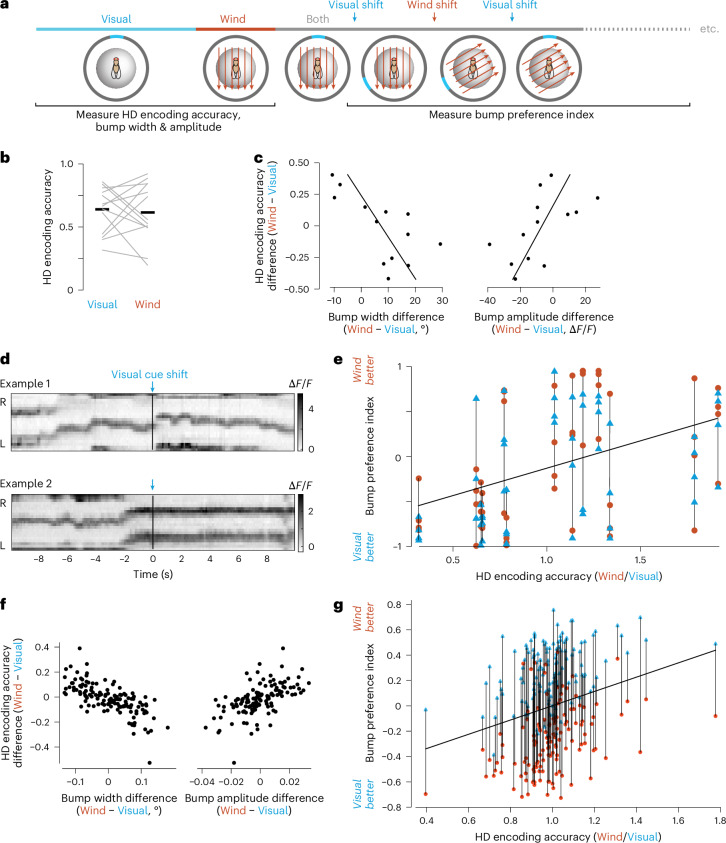
More informative cues are accorded more weight. This figure shows data for 13 flies. **a**, A bright visual cue and wind cue were tested individually and then combined. One cue was shifted 120° in an alternating sequence every 5 min. In some experiments, the visual cue came first (as shown here) while, in others, the wind came first. **b**, In the initial single-cue epochs, mean HD encoding accuracy is similar for the two cues (*P* = 0.76, paired two-sided Wilcoxon test). Thick horizontal lines are mean values. Gray lines are individual flies. **c**, In the initial single-cue epochs, the difference in HD encoding accuracy for the two cues is correlated with the bump width difference (*R*^2^ = −0.68, *P* = 0.01, Pearson correlation) and the bump amplitude difference (*R*^2^ = 0.58, *P* = 0.04, Pearson correlation). **d**, Example responses to +120° shifts of the visual cue. In example 1, the bump shifts upward to follow the upward (positive) shift of the cue, whereas, in example 2, it does not. In both cases, the wind does not shift. Extended Data Figure 3a shows these examples in more detail. **e**, The bump preference index is significantly correlated with relative HD encoding accuracy in the two single-cue environments (*P* = 0.03, Pearson correlation). Each gray line at a single encoding accuracy connects the data for one fly (four wind shifts in orange and four visual shifts in blue). An index of −1 means that the bump follows the visual cue, whereas +1 means that the bump follows the wind. This index is close to −1 for example 1 and close to +1 for example 2 (blue numerals). **f**,**g**, Same as **c** (**f**) and **e** (**g**) but for model networks with two populations of ER neurons. In different simulation runs, we varied the amplitude of sensory activity of one ER population while holding the amplitude constant in the other population.

Next, to investigate how different cues are integrated, we introduced wind into our virtual reality environments. We delivered wind through a tube that we rotated around the fly whenever the fly turned on the spherical treadmill, such that the environmental wind direction appeared constant from the fly’s perspective. At the outset of each experiment, we presented the visual cue and the wind cue alone (Fig. 4a) and we confirmed that they produced similar HD encoding accuracy on average (Fig. 4b). However, in some individuals, HD encoding accuracy was higher with the visual cue, whereas, in other individuals, HD encoding accuracy was higher with the wind cue. In general, the cue that generated better HD encoding accuracy was the cue that produced the narrower and higher-amplitude bump (Fig. 4c). Thus, one cue was often experienced as conveying more information, although the two cues were equally informative on average.

After we combined the cues, we shifted one or the other every few minutes to create a conflict between them and to see which cue carried more weight. We saw that the bump sometimes followed the shifted cue while, in other instances, it did not move (Fig. 4d). Across all trials and all individuals, visual shifts and wind shifts had a similar influence on the bump (Extended Data Fig. 3). Nonetheless, some individuals systematically gave more weight to the visual cue, meaning that the bump followed visual shifts more than wind shifts. In general, these were the individuals where the visual cue produced higher HD encoding accuracy when it was presented alone (Fig. 4e). Conversely, other individuals gave more weight to the wind; these were the individuals where the wind produced higher HD encoding accuracy when presented alone (Fig. 4e). In short, the cue that produced better HD encoding was generally accorded more weight in cases of cue conflict.

Our model can reproduce our results if we have two populations of ER neurons and we vary the ER amplitude of one or the other population. When each cue is presented individually, this produces correlated individual variations in HD encoding accuracy and bump width, as well as weak effects on bump amplitude (Figs. 2h and 4f). When the cues are presented together and one is shifted, we find that the relative HD encoding accuracy of the two cues is a good predictor of the bump preference index (Fig. 4g).

As an aside, we noticed that flies with visually biased HD systems sometimes showed clear behavioral reorientation into the wind after a wind shift (Extended Data Fig. 3). In these flies, the HD system evidently interprets the wind shift as a shift in the wind’s environmental direction, not a shift in HD; nonetheless, these flies still reorient into the wind after it shifts. Here, behavioral reorientation into the wind is likely mediated by pathways for orientation control that bypass the HD system^32,33,44^. These observations confirm that these flies are able to detect both cues and the findings are consistent with a model where individual biases originate with ER neurons, as ER neurons are specifically devoted to the HD system.

To summarize, we find that the same cue produces a more accurate HD encoding in some individuals than in others. This implies that the cue carries more or less information for different individuals. Furthermore, each individual accords more weight to the cue they experience as more informative. We can account for these results by positing individual variations in the amplitude of the sensory responses in ER neurons.

### Cue combinations change the bump profile and drive learning

Normally, the relationship between different cues should be relatively stable rather than constantly shifting. As the organism acquires more familiarity with a given cue, it should ascribe more weight to this cue, as long as the cue continues to appear stable. In principle, this should produce a more accurate HD system and the multisensory environment should be encoded more accurately.

**Fig. 5:**
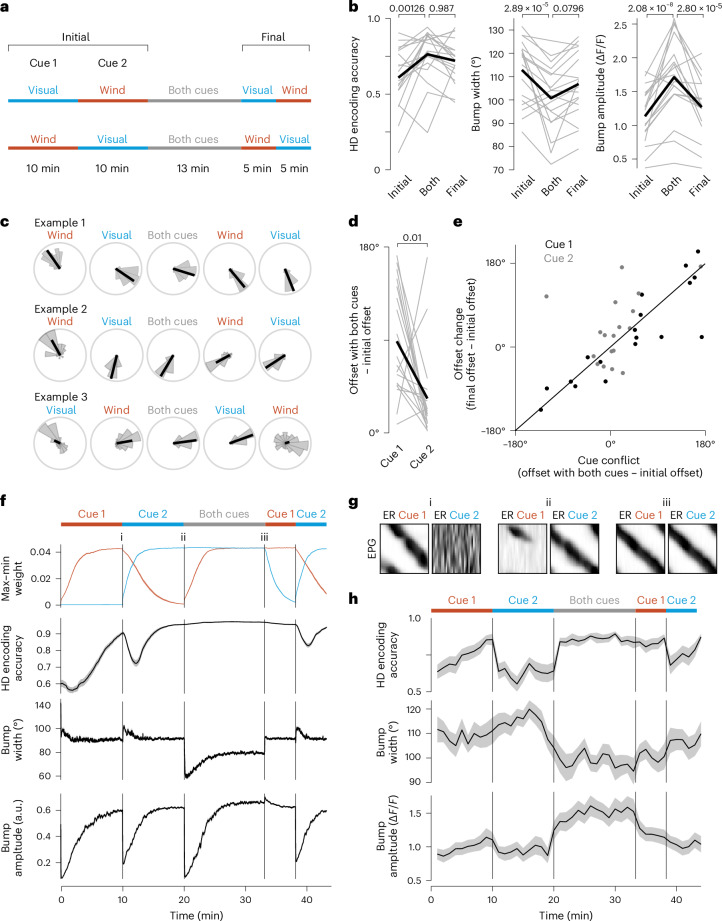
Cue combinations change the bump profile and drive learning. This figure shows data for 18 flies. **a**, Experimental protocol. Each cue was tested alone, then the two cues were combined and, finally, each cue was retested alone. The first cue was either the bright visual cue or wind. During the initial single-cue blocks, the two cues produce similar HD encoding accuracy and similar bump profiles, on average (Extended Data Fig. 4). **b**, HD encoding accuracy, bump width and bump amplitude for all three blocks; *P* values are for linear mixed-effects models with Tukey comparisons and Bonferroni corrections. **c**, Offset distributions for three example flies (Extended Data Fig. 4). **d**, Absolute difference between the offset with both cues and the initial offset. This difference is significantly smaller for cue 2, which is the more familiar cue (*P* value from paired two-sided Wilcoxon test). In most flies, cue 2 captures the bump during the epoch with both cues. **e**, Offset change (final − initial) versus offset conflict during the cue combination period (both − initial). There are two data points per fly, one for cue 1 (the less familiar cue) and the other for cue 2 (the more familiar cue). The line shown in the plot is a fit to all these points, based on the model that describes the offset change as a linear function of conflict with von Mises noise (Methods). **f**, Model showing the temporal evolution of max–min ER → EPG weight (that is, the range of weight values in the matrix), HD encoding accuracy, bump width and bump amplitude. **g**, Model showing ER → EPG weights at three time points (i, ii and iii). **h**, The same parameters tracked over time in the experimental data (mean ± s.e.m. across flies). Here, all parameters were measured in a rolling 60-s time window (Extended Data Fig. 5).

To test these predictions, we combined the two cues in a stable configuration after first testing them individually (Fig. 5a and Extended Data Fig. 4). We found that a stable configuration of these two cues increased HD encoding accuracy (Fig. 5b). This result confirms our expectation that the two cues together convey more information than either cue alone. Moreover, a stable configuration of these two cues also produced a narrower and higher-amplitude bump (Fig. 5b).

In the two-cue environment, we found that the bump generally retained its offset with respect to the cue that had been presented last (that is, the more recently familiar cue) (Fig. 5c,d). The more recently familiar cue was generally the dominant cue in the two-cue environment, regardless of whether that was the visual cue or the wind (Extended Data Fig. 4). Thus, when we moved the fly into the two-cue environment, its HD system continued to function seamlessly without a change in the representational coding frame.

When we retested each cue at the end of the experiment, we found that the system had reorganized to align the effects of the two cues. In general, the offset with respect to the less familiar cue (cue 1) changed dramatically, whereas the offset with respect to the familiar cue had changed much less. For example, in the first example shown in Fig. 5c, the wind and the visual cue have initial offsets almost 180° apart. Thus, when we place the visual cue in the upwind direction, the two cues are pushing the bump to opposite locations. In this fly, the visual cue was presented last and the bump retained its offset with respect to this cue. At the end of the experiment, we found that the wind offset had changed to match the visual offset.

Overall, we found that the change of each cue offset was predicted by the conflict during the two-cue epoch; here, we define conflict as the difference between the offset with both cues and the initial offset. Specifically, the bump offset often changed dramatically for the less familiar cue where the conflict was often large; conversely, the bump offset generally did not change for the more familiar cue where the conflict was generally small (Fig. 5e). These findings argue that the more familiar cue instructs the remapping of the less familiar cue to produce a self-consistent representation of the environment.

Our network model can explain all these results. When cue 1 appears, the Hebbian learning rule progressively etches a notch into the weights associated with cue 1 (Fig. 5f,g), which slowly increases HD encoding accuracy. We see the same slow increase in HD encoding accuracy in our data (Fig. 5h). Note that LTD here represents ‘learning’. Next, cue 2 appears and synaptic plasticity progressively etches a notch in the weights associated with cue 2; meanwhile, the notch associated with cue 1 is gradually erased, as LTP (‘forgetting’) outweighs LTD (learning) when the cue is absent. This is because LTP depends only on postsynaptic activity. Then, when cue 1 reappears while cue 2 is still present, cue 2 is now dominant, because its weight notch is deeper; in other words, the memory of this cue is stronger because it has been familiar more recently (Fig. 5f). In the two-cue environment, the bump is narrow because there is more inhibitory drive to the network. Over time, synaptic plasticity recreates a notch in the weights associated with cue 1 but, now, the Hebbian learning rule ensures that the two notches are well aligned, which explains the offset changes we observed in our data (Fig. 5d,e). This causes the active EPG neurons to be even more disinhibited than they were previously, meaning that bump amplitude increases (Fig. 5g). Subsequently, cue 2 disappears but HD encoding accuracy remains high because cue 1 has been well learned (highly familiar) by this point; this is also a phenomenon we observe in our data (Fig. 5h). This ‘priming’ effect in the model is because of Hebbian plasticity. This priming effect can account for the observation that a salient cue can persistently increase HD encoding accuracy even after that cue is removed^42^. Lastly, the return to cue 2 produces a drop in HD encoding accuracy because cue 2 has already been partially forgotten.

To summarize, our data show that cue combinations increase HD encoding accuracy, narrow the bump, increase bump amplitude and trigger learning. When two cues conflict, the more familiar cue is weighted more heavily and used to guide the remapping of a less familiar cue. All these findings can be explained by a ring attractor model with highly plastic sensory synapses. In essence, Hebbian plasticity stores the image of a familiar cue and subsequently uses this image to instruct a new round of plasticity at the synapses associated with the less familiar cue. Although we cannot observe synaptic weights in our experimental data, we can infer the pattern of weights by tracking the position and amplitude of the EPG activity bump; specifically, the bump’s position (relative to a cue) tells us the location of the notch in the weight matrix, while the bump’s amplitude tells us about the notch depth.

### A cue that produces a wide bump is remapped more readily

There is a basic tradeoff between stability and flexibility in any representation of the environment; when the representation is stable, it is accurate but this makes remapping more difficult. In particular, if the HD system ascribes too much weight to any particular cue, it may be difficult to learn a new interpretation of that cue. We can state this idea in mechanistic terms; if some sensory connections onto HD cells become dominant, then other inputs cannot compete with them and their weights cannot change. Therefore, a cue that is weighted heavily may be difficult to remap after the environment changes.

**Fig. 6:**
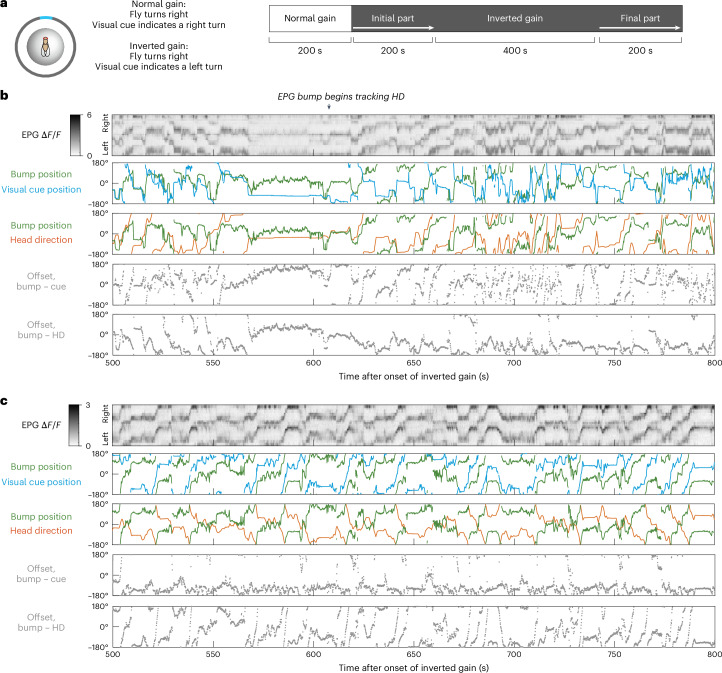
Learning to invert the mapping of a visual cue. **a**, Schematic of the protocol. **b**, Data from an example experiment, shown for the last 300 s of the inverted gain block. The EPG bump begins to track the fly’s HD (arrowhead). **c**, Data from another example experiment. In this case, the HD system simply follows the visual cue for the entire inverted gain block and, thus, it does not accurately track HD.

To test this prediction, we challenged flies to learn an inverted gain in virtual reality, such that the visual cue moves in the ‘wrong’ direction whenever the fly rotates (Fig. 6a). This is a dramatic change in the environment, which produces a strong conflict between the visual cue and the self-motion input to the HD system. In principle, it might seem that the HD system should simply ignore the visual cue in this situation but this would lead to poor HD encoding accuracy because this system requires feedback for accurate performance (Fig. 2c). Therefore, the network should ideally learn to invert the mapping of the visual cue onto the EPG cell ensemble. A previous study reported that an optogenetic method could be used to artificially create this type of inversion^22^ and this motivated us to investigate whether it was possible to obtain the same type of inversion through visuomotor learning.

Notably, we found that some individuals were able to learn to invert the mapping of the visual cue. In these individuals, bump dynamics were generally unpredictable during the initial part of the inverted gain experience; however, at some point, the bump would begin tracking HD fairly accurately (Fig. 6b). By contrast, other individuals never learned to invert their interpretation of the visual cue. In these cases, the bump generally just tracked the visual cue; thus, when the fly turned right, the HD system registered a left turn (Fig. 6c).

**Fig. 7:**
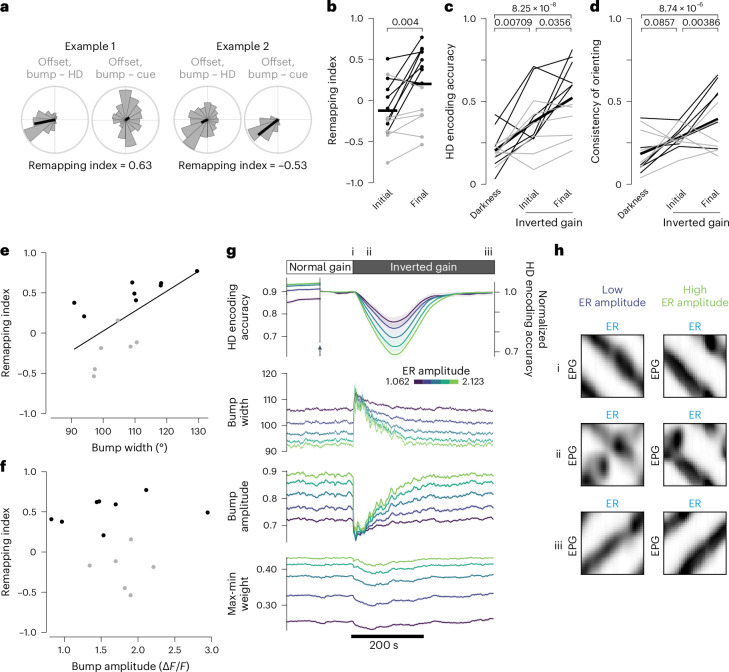
Bump width in normal gain predicts cue remapping in inverted gain. Panels **b**,**e**,**f** show data from 14 flies, while **c**,**d** show data from 12 flies because two flies did not receive a ‘darkness’ trial. **a**, Offset distributions for the two example flies from Fig. 6. The remapping index is positive for fly 1 because the offset with respect to HD is more stable. The remapping index is negative for fly 2 because the offset with respect to the visual cue is more stable. **b**, Remapping index in the initial and final part of the inverted gain block. Across all flies, the remapping index is larger in the final part (*P* value from two-sided Wilcoxon test). Black and gray lines denote individuals with final remapping indices above or below the mean (horizontal bar); this is preserved in **c**–**f**. **c**,**d**, HD encoding accuracy (**c**) and consistency of behavioral orientation (**d**) in darkness versus inverted gain (*P* values from linear mixed-effects models with Tukey comparisons and Bonferroni corrections). **e**,**f**, Final remapping index is predicted by bump width (**e**; *P* = 0.02, Pearson’s correlation) but not bump amplitude (**f**; *P* = 0.79, Pearson’s correlation) during the preceding normal gain block (*n* = 14 flies) (Extended Data Fig. 6). **g**, Model showing the effect of varying the amplitude of visual responses in ER neurons. Lower ER amplitude produces a faster recovery of HD encoding accuracy after the onset of inverted gain. Note that lower ER amplitude produces lower overall HD encoding accuracy; to facilitate a comparison between conditions, we show the normalized HD encoding accuracy (normalized to 1 at end of normal gain epoch) beginning halfway through the normal gain epoch (arrow). Results are shown as the mean ± s.e.m. across 100 simulation runs. Panel **h** shows weight matrices for the indicated time points (i, ii and iii). **h**, Model showing ER → EPG weights at the end of the normal gain block (i), soon after the onset of inverted gain (ii) and when remapping is nearly complete (iii). Results are shown for two ER amplitudes (1.327 and 2.123). At the intermediate time point (ii), remapping has progressed further with the lower ER amplitude.

To quantify learning, we defined a remapping index, where +1 means that the bump is correctly tracking the fly’s rotation, implying successful remapping; conversely, −1 means that the bump is moving against the fly’s rotation, implying no remapping (Fig. 7a). On average, the remapping index was significantly higher in the final part of the inverted gain block as compared to the initial part (Fig. 7b). Moreover, during the inverted gain block, the mean HD encoding accuracy increased (Fig. 7c) and the consistency of behavioral orienting also increased (Fig. 7d). Thus, spatial learning was clearly occurring during the inverted gain block.

Importantly, the individuals with high remapping indices were not simply learning to ignore the visual cue because their HD representation was much more stable than it would be in darkness. In darkness, HD encoding accuracy was always low (Fig. 2c), whereas HD encoding accuracy was relatively high by the final part of the inverted gain block, at least in some individuals (Fig. 7c). Thus, these individuals are not learning to discount the cue; rather, they are clearly learning to invert their interpretation of the cue.

We could predict which individuals would be successful at learning this inversion on the basis of the profile of the EPG bump in the normal gain block before the onset of inverted gain. Specifically, we found that individuals with the widest bumps (in the normal gain block) all had relatively high remapping indices at the end of the inverted gain block (Fig. 7e). In other words, a wider bump was predictive of better remapping. There was also a trend for these same individuals to have lower HD encoding accuracy during the normal gain block, consistent with their wider bumps, although this fell just short of statistical significance (Extended Data Fig. 6). Bump amplitude in the normal gain block was not correlated with remapping (Fig. 7f).

Similarly, we found that our network model could also learn to invert its interpretation of the visual cue. This works because self-motion cues instruct Hebbian learning at ER → EPG synapses; thus, ER neurons can learn to push the bump in whatever direction is consistent with self-motion signals. In the model, we can obtain individual variations in performance by varying the amplitude of visually evoked activity in ER neurons (Figs. 3 and 4). We found that a lower ER amplitude allows faster remapping (Fig. 7g) because self-motion cues have more weight relative to the visual cue. This allows self-motion cues to better instruct the appropriate reinterpretation of the visual cue, thus more rapidly reversing the orientation of the notch in the ER → EPG weight matrix (Fig. 7h). A lower ER amplitude also produces a wider bump (Fig. 7g), which explains why a wider bump predicts faster learning (Fig. 7e). In the model, a lower ER amplitude also produces a smaller bump amplitude, which was not correlated with learning in our data; this may reflect our limited sample size or a real difference between the model and the actual network.

In summary, we found that, when a cue produces a wider bump, it is remapped more readily after a switch to inverted gain. We can reproduce this result in a network model where we vary the amplitude of the sensory inputs to the HD network. In this model, individuals that experience the cue as being less intense are better able to remap that cue after a switch to inverted gain. This result highlights a basic tradeoff between stability and flexibility in this system; when a spatial representation is highly stable, it is highly accurate but this also makes it more difficult to adjust this representation when conditions change.

## Discussion

There is a tradeoff between stability and flexibility in neural network dynamics. On one hand, theoretical attractor networks are often tuned to be stable, such that the bump of activity in the network hardly varies in amplitude or width^28,45,46^; these networks are designed to support working memory but not learning. Theoretical attractor networks are sometimes also assumed to have fixed synaptic weights at their sensory inputs to minimize representational drift^47,48^. On the other hand, the biological attractor networks that underpin the brain’s navigational brain regions^17^ likely support learning about the relative positions of environmental cues and the stability of those cues. Thus, as cues change and learning proceeds, we might expect to see changes in synaptic weights^20–28^ and resulting changes in profile^49,50^. We do not have a general understanding of how biological neural networks manage the tradeoff between stability and flexibility.

In the *Drosophila* HD system, the bump of activity has a stable position when the fly is not moving, effectively storing a working memory of the fly’s orientation, using a ring attractor network. Here, we show that this bump varies systematically in width and amplitude depending on the intensity and familiarity of the cues in the environment. The changes we observe in the bump profile can be explained, in part, by changes in synaptic weights at sensory synapses onto HD cells. Although we cannot observe these synaptic weights directly, we can ascertain something about these weights on the basis of observed changes in bump profile. For example, when cue intensity increases or a cue becomes more familiar, we find that the bump becomes narrower and this can be at least partly explained by a deeper notch in the synaptic weight matrix. Moreover, we show that increases in cue intensity and familiarity produce increases in HD encoding accuracy and the consistency of behavioral orientation; thus, a more intense and familiar cue is actually more informative. When we then introduce conflicts between cues, we find that the cue that produces higher encoding accuracy is generally accorded more weight and this is predicted by the bump profile in response to that cue. Moreover, the cue that produces higher encoding accuracy generally instructs spatial learning in response to the cue conflict, which resolves the conflict and increases encoding accuracy. Together, these results provide concrete evidence for the theoretical proposal^20,21,26–31^ that Hebbian plasticity can endow an attractor with the flexibility it needs to support learning about the relative positions of environmental cues and the stability of those cues.

The idea that spatial learning might involve synaptic plasticity is intuitive but it is technically difficult to observe synaptic weights evolve in real time in a biological network. Here, we took the bump profile as a proxy for synaptic weights and showed that it takes minutes to achieve restabilization of the bump profile after a change in the environment; this result implies that it also takes minutes to achieve restabilization of synaptic weights. This timescale may represent a compromise between the demands of flexibility and stability; a faster timescale would accelerate learning but it would also accelerate forgetting when a cue transiently disappears and increase representational drift. In the *Drosophila* HD system, dopamine controls the learning rate and dopamine increases with exploratory movements^24^; this provides a mechanism to increase network flexibility during exploration, conversely allowing more stability at other times.

It is less intuitive to think that synaptic plasticity can help cues to be weighted by their informativeness. Accurate inference requires that more informative cues should be weighted more heavily and, in some cases, this reweighting process can occur instantaneously; for example, we immediately ascribe less weight to a visual cue when it is blurred or degraded^51,52^. In these cases, there is no need for inference to involve neural plasticity. However, in other cases, the informativeness of a cue cannot be assessed immediately; for example, during navigation, the informativeness of a visual cue depends on its stability within the environment, which can only be evaluated by learning. Hebbian plasticity has been suggested to assist inference under these conditions^21,25,29–31^ and our work provides direct support for this suggestion.

Importantly, our model can explain many features of animal navigation behavior. For example, navigating animals assign more weight to spatial cues that are more salient or more stable and familiar; this is true in both insects^8–13^ and vertebrates^3–7,14^. Our results imply that these types of cues can create stronger spatial modulations in synaptic weights from sensory cells. Previous work has also demonstrated that insects can form arbitrary learned associations between the angular positions of the wind and the sun^8,9,27^. Our results show how an arbitrary cue configuration can be stored in the pattern of synaptic weights from sensory cells.

Our results also provide insight into mechanisms underlying individual differences in navigation behavior^53,54^. Specifically, we showed that different individuals have different levels of encoding accuracy in the same virtual reality environment. Moreover, individuals with higher accuracy had significantly narrower bumps of activity. On the other hand, individuals with wider bumps were better able to reorganize their HD representations when the environment changes. In our model, we could recapitulate all these individual differences by varying the amplitude of sensory inputs to the HD system. Importantly, individual variation is not specific to flies; human subjects also show marked idiosyncratic differences in cue weighting during navigation^14,55–58^. Individual differences may reflect variations in each individual’s past experiences. Alternatively, they may reflect an evolutionary strategy; for example, it might be useful for the species if some individuals have more stable mental representations of space (sacrificing flexibility), while others have more flexible representations (sacrificing stability).

The fact of individual differences in navigation should make it obvious that navigation is not always optimized. Although the model network we describe here has some remarkable features, it cannot perform optimal Bayesian inference, even in the absence of these individual differences. Optimal inference would require the network to store the certainty associated with the network’s HD estimate, for example through some mechanism of persistent activity that boosts bump amplitude after an informative cue is presented, such that bump amplitude remains persistently high for some time after the cue disappears^49,50^. Our data imply that this does not occur (Extended Data Fig. 8); accordingly, our model network in this study does not store certainty about HD, instead storing information about the salience, stability and familiarity of each cue. Curiously, there are hints that the insect brain does have a way to represent certainty during navigation. For example, homing ants will search for their nest over a wider radius if they have just returned from a longer trip, which suggests that they keep track of their certainty and use this to adjust their search strategy^59^. Moreover, ants will steer further downwind of their expected nest site when they have returned from a longer trip, which should help them to use odor filaments as a guideline back to their nest when their certainty is low^60^. In the future, it will be interesting to investigate whether these behaviors actually arise from a neural representation of certainty or whether they reflect simpler behavioral strategies^27^.

## Methods

### Experimental model and subject details

*Drosophila melanogaster* were raised on cornmeal and molasses (Archon Scientific) under a 12-h light–dark cycle at 25 °C. Experiments were performed on 1-day-old virgin females with the genotype *w/+;+;P{R60D05-Gal4}attP2/P{20XUAS-IVS-jGCaMP7f}VK00005*. Both *P{R60D05-Gal4}attP2* and *P{20XUAS-IVS-jGCaMP7f}VK00005* were obtained from the Bloomington *Drosophila* Stock Center (RRID:BDSC_39247 and RRID:BDSC_79031, respectively). *P{R60D05-Gal4}attP2* drives Gal4 expression in EPG neurons, as reported previously^34,41^, and its construction was described previously^61^. *P{20XUAS-IVS-jGCaMP7f}VK00005* was also described previously^40^.

### Fly selection and housing

Virgin female flies were anesthetized on CO_2_, collected at least 12 h before the experiment and then allowed to recover on molasses food. Imaging experiments were conducted the following day, 14–36 h after eclosion. Before the experiment, flies were starved for 0–24 h on a piece of damp laboratory tissue (Kimtech). The starvation time was chosen on the basis of our observations for what resulted in the best fly behavior during the months in which each experiment was conducted (Figs. 2 and 3 and Extended Data Figs. 2, 5, 7 and 8: 5–24 h; Fig. 4 and Extended Data Fig. 3: 0 h; Fig. 5 and Extended Data Fig. 4: 0–3 h; Figs. 6 and 7 and Extended Data Fig. 6: 18–27 h). No statistical methods were used to predetermine sample sizes but our sample sizes are similar to those reported in previous publications^23,24,33,34,37,38^.

### Fly preparation and dissection

Flies were briefly cold-anesthetized in a glass vial (V7005-500EA, Sigma-Aldrich) on ice and placed inside a custom-made inverted pyramidal platform CNC machined from black Delrin (Protolabs Inc.). The head was tilted forward to make the posterior part of the brain more accessible during imaging. Because of the head’s angle and the pyramidal shape of the holder, the majority of each eye was positioned below the holder and, therefore, able to see the visual stimuli. The wings were removed and the head and thorax were secured to the holder using ultraviolet-curable glue (Loctite AA 3972) and cured with ultraviolet light (LED-200, Electro-Lite). To prevent large brain movements, the proboscis was removed (we again briefly cold-anesthetized the animal during this process). The head was then bathed in an extracellular saline solution with the following composition: 103 mM NaCl, 3 mM KCl, 5 mM TES, 8 mM trehalose, 10 mM glucose, 26 mM NaHCO_3_, 1 mM NaH_2_PO_4_, 1.5 mM CaCl_2_ and 4 mM MgCl_2_ (osmolarity 270–275 mOsm, bubbled with 95% O_2_ and 5% CO_2_, to reach a final pH of ~7.3). A window was opened in the head cuticle and the trachea and fat were removed to better expose the brain. To reduce additional brain movements, muscle 16 was clipped.

### Two-photon calcium imaging

We performed in vivo calcium imaging with a two-photon laser scanning microscope with a galvo-resonant scanner (Bergamo II, Thorlabs). We used a femtosecond Ti:sapphire laser with dispersion precompensation (Vision-S, Coherent) tuned to 940 nm to achieve two-photon excitation. To image, we used a ×20 objective (numerical aperture: 1.0; XLUMPFLN, Olympus) mounted on a fast objective scanner (P-725, Physik Instrumente). The emission from our samples was detected with a GaAsP photomultiplier tube (PMT) detector (Hamamatsu) equipped with a 525-nm bandpass filter (Thorlabs). We collected imaging data using National Instruments PXIe-6341 hardware with ScanImage^62^ 2018b or 2020 (Vidrio Technologies, RRID:SCR_014307). We defined an image as 256 × 128 pixels encompassing the protocerebral bridge (PB). We acquired a volume of 12 slices, with 5-μm steps separating consecutive slices, at a rate of 9.18 volumes per second. We discarded the slices corresponding to ‘flyback’ frames post hoc.

### Fly locomotion

The platform holding the fly was positioned above a spherical treadmill, consisting of a 9-mm-diameter ball made of foam (FR-4615, General Plastics). The ball was floated on a steady stream of medical grade breathing air at ~0.2–0.3 L min^−1^ in a custom holder three-dimensionally (3D) printed using Grey Pro v2 resin (Formlabs). An irregular black pattern was painted on the ball (Vallejo Black Model Color Paint) to allow tracking of the ball surface with machine vision. The fly was positioned on the ball under visual control, using a side camera (CM3-U3-13Y3M-CS, forward-looking infrared (FLIR); InfiniStix lens 94 mm, ×0.50, Infinity Photo-Optical) and a front camera (BFS-U3-13Y3M, FLIR; InfiniStix lens 94 mm, ×0.50, Infinity Photo-Optical). The ball was illuminated with a round board of 36 infrared light-emitting diodes (LEDs) (SODIAL). The image from one of the cameras was acquired at 50 Hz and analyzed using FicTrac^63^ version 2.1.1 (rjdmoore.net/fictrac/) to track the position of the ball in the pitch, yaw and roll axes, thereby reconstructing the fly’s locomotor trajectory. This camera was positioned at an angle to ensure that a sufficient fraction of the ball surface was always visible, avoiding occlusion by the rotating air nozzle used to deliver the wind.

### Visual stimuli

Visual stimuli were displayed on a custom-built cylindrical panorama of LEDs adapted from published prototypes^64^. The panorama covered the entire 360° range of azimuthal angles. It consisted of two rows and 12 columns of square blue LED panels (peak: 470 nm), with each panel consisting of 8 × 8 pixels. One panel was removed in the top row (on the fly’s right) to accommodate the side camera used for fly positioning and ball tracking. The visual arena was tilted forward to match the inclination of the fly’s head in the platform.

The LED panels were covered with a diffuser material (SXF-0600, snow-white light diffuser, Decorative Films). Moreover, five layers of gel filters were used to reduce overlap in spectra and to decrease the intensity of the stimuli: three layers of Tokyo blue (Rosco, RE071), one layer of 0.3 neutral density (Rosco, RE209) and one layer of marine blue (Rosco, RE131). Additionally, the back, top and bottom of the panorama were covered with black tape to further reduce the amount of LED light reaching the PMTs.

The visual cue consisted of a blue vertical stripe (two pixels wide, 7.5°), spanning the vertical extent of the panorama. Visual stimuli were programmed in Matlab 2020a (MathWorks, RRID:SCR_001622). Custom Python software was used to read FicTrac outputs and generate analog voltage signals through a Phidget analog output device (Phidget Analog 4-Output 1002_0B). For closed-loop control of the stimulus, the ball displacement in the yaw axis was used to update the azimuthal position of the visual cue (refresh rate ≥ 372 Hz). Analog output signals from the visual panel system were digitized with a NI-DAQ PCI-6351 (National Instruments) at 4 kHz. The intensity of the background was 0/15. The bright cue had an intensity of 15/15, whereas the dim cue had an intensity of 1/15. These intensity values were chosen empirically on the basis of the results of pilot experiments, to ensure that HD encoding accuracy was lower for the dim cue versus the bright cue, but HD encoding accuracy was still higher for the dim cue than in conditions of darkness. We also deliberately chose values of cue brightness and wind speed (discussed below) such that the bright cue and the wind were roughly equally informative, meaning that they produced similar values of HD encoding accuracy (averaged across flies).

### Wind stimuli

Wind stimuli were delivered using a custom-built device that is conceptually similar to a previously published device^65^. Our device uses a commutator to maintain the air flow as the nozzle rotates around the fly in 360°. The commutator was 3D-printed from Rigid 4000 resin (Formlabs) in two pieces. The base piece had a ball holder and two air intake ports (one for the air-supported ball and another for the wind delivery). The top piece had an air nozzle (inner diameter: 2.8 mm) that rotated around the fly. When the nozzle was in front of the fly, it was positioned 10 mm from the antennae. A ball bearing (McMaster-Carr, 5908K19) was used to create a smoothly rotating interface between the top and bottom pieces. To rotate the air nozzle, a timing belt (0.25-inch width; McMaster-Carr, 6484K118) was attached to the top part of the commutator and connected to a pinion pulley (Servocity SKU, 615424), which was mounted on the shaft of a stepper motor (Pololu, 1204). The entire wind delivery device was designed such that it would fit inside the 360° visual panorama described above.

The air nozzle was printed from black material (Black Resin, Formlabs) to reduce its visibility. Control experiments were performed to ensure that the nozzle did not interfere with the fly’s ability to see the LED arena and that the nozzle itself was not acting as a visual cue. In these control experiments, each fly received three stimulus blocks: a block in a closed loop with a high-contrast visual cue, a block in a closed loop with wind and a block in a closed loop with ‘wind’ but with the air turned off (meaning that the nozzle moved around the fly as normal but no air was flowing). We found that the bump had a consistent offset relative to the virtual environment in the first two blocks (as expected) but it drifted relative to the environment in the third block (as we would expect from a fly walking in darkness); in other words, HD encoding accuracy was high in the first two blocks and low in the third block. This result confirmed that the visual image of the nozzle did not act as an effective HD cue.

Wind direction was controlled using Python and Arduino. The stepper motor was controlled using a controller board (X-NUCLEO-IHM02A1, STMicroelectronics) and Arduino UNO. Arduino code allowed us to specify the location of the air nozzle in the next time step and a custom Python code communicated with the Arduino through serial port to generate the pattern of nozzle movements. When the wind was in closed loop with the fly’s rotation, the Python code obtained the current HD of the fly through FicTrac (as described above) and generated the command to move the air nozzle in the appropriate location, such that the allocentric wind direction remained constant, from the fly’s perspective.

The wind speed was 0.2 m s^−1^, measured with a hot-wire anemometer (A004, Kanomax) at the fly’s location. Air flow was regulated through a mass flow controller (Aalborg, GFC17A-VAL6-C0). Before each experiment involving wind stimuli, we confirmed that the air nozzle was accurately pointing at the fly’s antennae by observing the movement of the aristae in response to wind using a camera (BFLY-PGE-31S4M, FLIR) equipped with a high-magnification lens (InfiniStix lens 44 mm, ×3.00, Infinity Photo-Optical).

### Stimulus protocols

For Figs. 2 and 3 and Extended Data Figs. 2 and 8, flies were in a closed loop for 20 min with a stimulus that switched across three contrast levels in 200-s blocks. The bright cue was a bright stripe (brightness level 15/15) against a black background (0/15). The dim cue was a dim stripe (1/15) against a black background (0/15). In the no-cue condition, the panorama displayed a black background (0/15). The block sequence was drawn randomly for each fly. Figure 2 uses data from all blocks, while Fig. 3 focuses only on each fly’s first block with the dim cue, because that was the block where HD encoding accuracy was most variable across individuals. Extended Data Fig. 2 uses data from the first block of each type. Extended Data Fig. 8 uses data from the transitions between darkness and the bright cue.

For Fig. 4 and Extended Data Fig. 3, flies were first in a closed loop for 10 min with a single cue: a bright stripe (15/15) against a dark background (0/15) or wind. Then, flies were in a closed loop for 5 min with the cue they did not receive in the first block. Next, the flies were in a closed loop with both cues for 45 min. Every 5 min, one of the cues shifted, with alternating visual shifts and wind shifts. If the fly had been presented with a visual cue as the first stimulus, then the visual cue was the first cue to jump and vice versa. Cue shifts were +120° or −120°, with the direction determined randomly for each shift. Because of the nature of our stimulus delivery, the visual cue shift occurred essentially instantaneously but the wind shift was limited by the stepper motor speed, taking ~0.4 s to rotate to its new location.

For Fig. 5 and Extended Data Fig. 4, flies were first in a closed loop for 10 min with a single cue: a bright stripe (15/15) against a black background (0/15) or wind. Then, flies were in closed loop for 10 min with the cue they did not receive in the first block. The block order (visual then wind or wind then visual) was drawn randomly for each fly. Next, flies were in a closed loop with both cues presented simultaneously for 800 s. In the fourth block, flies were again in a closed loop with the initial single cue, this time for 5 min. Finally, flies were in a closed loop with the second single cue, also for 5 min.

For Figs. 6 and 7 and Extended Data Fig. 6, flies were first in a closed loop with a bright stripe (15/15) for 15–20 min to allow the compass system to stabilize; data from this stabilization epoch are not shown in the figures but instead used for Extended Data Figs. 5 and 7 (top). Next, flies were in a closed loop with a bright stripe for 20 min. For the first 200 s, the gain between the fly’s movement and the cue movement was set to 1 (normal gain); for the next 800 s, it was set to −1 (inverted gain); for the final 200 s, it was reverted back to 1. Finally, flies walked in darkness for 200 s.

For Extended Data Figs. 5 and 7 (top), flies were in closed loop with a bright stripe (15/15) against a black background (0/15) for 15–20 min.

For Extended Data Fig. 7 (bottom), these panels show responses to an open-loop presentation of a rotating visual cue. The cue was a bright stripe (15/15) against a black background (0/15). Each fly received 14 60-s blocks, with the cue rotating at 30°, 60°, 90°, 120°, 150°, 180° or 200° per second (two blocks per stimulus speed, with speed order randomized).

#### Randomization and blinding

The experimenter was not blind to the experimental conditions assigned to each fly. Blinding is only potentially relevant to experiments where different flies experienced different experimental treatments (for example, different genotypes or different drug treatments). However, there was only one experiment where different flies experienced different experimental treatments, namely the cases where the order of the different brightness blocks was randomized (Figs. 2 and 3 and Extended Data Figs. 2 and 8). Here, the experimenter could not be blinded to the experimental condition because the stimulus apparatus delivered the stimulus in a manner that was visible to the experimenter.

### Data analysis

Data analysis was performed using Matlab R2021a (MathWorks, RRID:SCR_001622), Python 3.7 and 3.9 (www.python.org/, RRID:SCR_008394), Stan^66^ (mc-stan.org/, RRID:SCR_018459), PyStan 2.19.0 (pystan2.readthedocs.io/en/latest/), R 4.1.3 (www.r-project.org/, RRID:SCR_001905) and RStudio 2022.02.0 (www.rstudio.com/, RRID:SCR_000432). Tukey tests and Pearson correlations assumed that data were distributed normally but this was not formally tested. No flies were excluded from analyses.

#### Preprocessing imaging data

**Extended Data Fig. 1:**
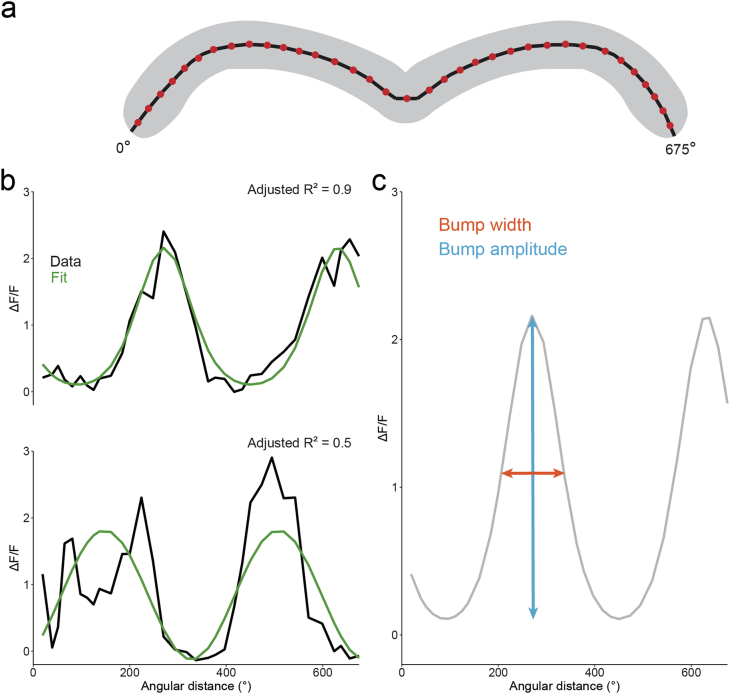
Fitting the bump of activity in the HD system. **a**. Schematic illustrating procedure for dividing the PB into spatial bins. First, the maximum *z*-projection was used to draw a mask around the protocerebral bridge (gray). Then, the *drawpolyline* function in Matlab was used to trace a line (black) composed of many short segments (25-40 segments) running along the midline of the PB. Each segment defines a spatial bin (that is,a region of interest or ROI). Next, a custom algorithm was used to find the line bisecting each segment perpendicularly (a normal line), and then to find the shortest distance between each pixel in our PB mask and one of the normal lines. The signal from each pixel is then assigned to the ROI corresponding to that normal line, with the entire array of ROIs representing an angular distance of 675°, that is one complete HD representation (360°) plus 7/8 of a second representation (315°)^41^. Note that previous studies have divided the PB into 16 spatial bins^37,38^, but here we use many more spatial bins (up to 40). This is justified because the fluorescence emission from each EPG neuron will tend to “spill out” from its home glomerulus, due to (1) the scattering of fluorescence emission into adjacent glomeruli, and (2) the presence of stray EPG axonal branches in adjacent glomeruli. As a result of this spillover, fluorescence signals are not perfectly compartmentalized within glomeruli. Thus, dividing the protocerebral bridge into more spatial bins should improve our ability to resolve the amplitude and shape of the bump of activity. Indeed, we found that this approach produced a more reliable measure of bump width, as indicated by higher variance explained (adjusted R^2^) when fitting linear models to bump width data (as in Figs. 3, 4, 7 and Extended Data Figs. 2, 5, 7). **b**. ΔF/F and fitted function for two example time points. Shown are a case with very good goodness of fit (top), and a case with a borderline fit quality. In the latter case, adjusted R^2^ = 0.5, which is our threshold for discarding data points from the analysis. **c**. Fit from the top example in panel b, showing the two parameters that we extract from the fit.

**Extended Data Fig. 2:**
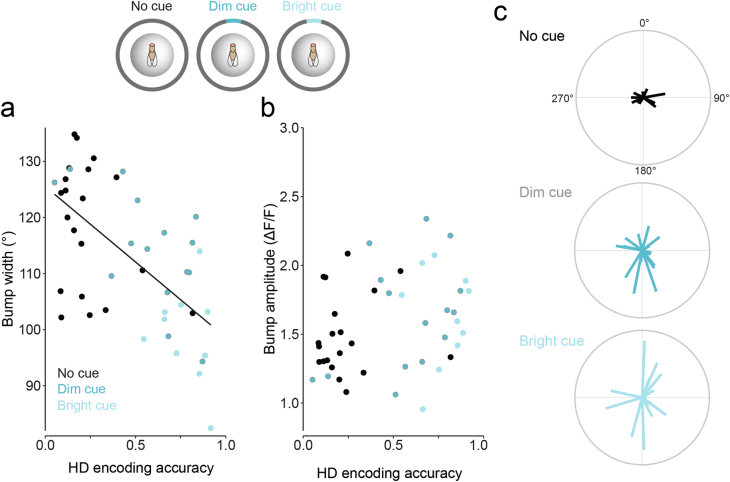
Individual variation in responses to cue brightness. This figure provides additional detail related to the 15 flies in Fig. 3. **a**. Variations in HD encoding accuracy correlate with bump width, pooling data across all three cue conditions and all 15 flies (p < 0.001, Pearson’s correlation). Gray points (dim cue) are reproduced from Fig. 3c. Black and blue points show data from the other two conditions (no cue and bright cue). **b**. There is a trend for variations in HD encoding accuracy to be correlated with bump amplitude, but the relationship falls just short of significance (p = 0.06, Pearson’s correlation). **c**. Mean head direction for each individual for all three cue conditions. The length of each line represents vector strength (that is, “consistency of behavioral orientation”, or 1-circular variance). Note that consistency of behavioral orientation increases with increasing cue brightness. Different flies choose different goal directions, which is typical of navigation driven by the HD system^32,33,42^.

The NoRMCorre algorithm^67^ (https://github.com/flatironinstitute/NoRMCorre) was used to perform rigid motion correction in the *x*, *y* and *z* dimensions. Then, the maximum *z*-projection was used to select a mask around the PB and define our region of interest (ROI). The PB was divided along its horizontal axis into 25–40 ROIs (Extended Data Fig. 1). The change in fluorescence ($\varDelta F/F$) was computed for each ROI, with the baseline fluorescence *F* defined as the bottom tenth percentile of fluorescence values for the trial for that ROI.

#### Analysis of locomotion data

The position of the ball in all three axes was computed by FicTrac at 50 Hz. This was used to infer the position of the fly in fictive two-dimensional space. Position data were unwrapped and then smoothed using locally weighted scatter plot smoothing (LOWESS smoothing). The velocity was computed in all three axes and smoothed again with the LOWESS method. The smoothed data were then downsampled to match the imaging volume rate.

#### Bump width and amplitude

A von Mises function of the form $f(x) = \alpha \,\exp (\kappa\,{\mathrm{cos}} (x-\mu ))+c$ was fit to each time point of our $\varDelta F/F$ signal with Matlab’s ‘fit’ function using the nonlinear least squares method and the trust-region algorithm for optimization. The estimated value of *μ* represents the bump’s position. The fit coefficients were then used to compute the bump width and amplitude:

$${\rm{bump}}\; {\rm{width}}=2\left|{\mathrm{cos}}^{-1}\left[\frac{1}{\kappa }\log \left(\frac{1}{2}\left({\exp }{(\kappa) }+{\exp }{(-\kappa })\right)\right)\right]\right|$$

$${\rm{bump}}\; {\rm{amplitude}}=a\left({\mathrm{exp}}{(\kappa) }-{\exp }{(-\kappa) }\right)$$

A goodness-of-fit metric (adjusted *R*^2^) was obtained for each time point and time points were discarded from group analyses if the adjusted *R*^2^ was below 0.5. Extended Data Figure 1 shows examples of a good fit and a poor fit.

#### HD

HD was taken as the time integral of the fly’s rotational velocity on the spherical treadmill, relative to the position with the cue directly in front of the fly (0°). In the two-cue environment in Fig. 5, the wind tube was aligned with the visual cue; thus, HD = 0° corresponded to the position where both the wind tube and the visual cue were directly in front of the fly. The bump position rotates clockwise in the ellipsoid body (EB; imaged from the posterior side of the head) as HD rotates counterclockwise; therefore, to account for this directionality when we plotted HD over time (for example, in Fig. 1f and elsewhere), we always plotted (−HD) to make it easier to visualize the correspondence between bump position and HD.

#### Offset and HD encoding accuracy

The offset of the bump relative to HD was computed as the circular distance between bump position and HD. Bump position rotates clockwise in the EB (imaged from the posterior side of the head) as HD rotates counterclockwise; therefore, to account for this directionality, we used (−HD) rather than HD:

$${\rm{offset}}={\rm{bump}}\; {\rm{position}}-(-{\rm{HD}})$$

In a single-cue environment, we defined HD = 0° as the value of HD that places the cue directly in front of the fly; thus, an offset of +90° means that the bump is positioned at +90° (on the right-hand edge of the EB) when the fly is facing the cue. To compute HD encoding accuracy, each offset value was treated as a unit vector and the vector strength of these values was calculated. HD encoding accuracy was computed only over the time points when the fly was moving; that is, moments of immobility were excluded.

#### Bump preference index and behavioral preference index

**Extended Data Fig. 3:**
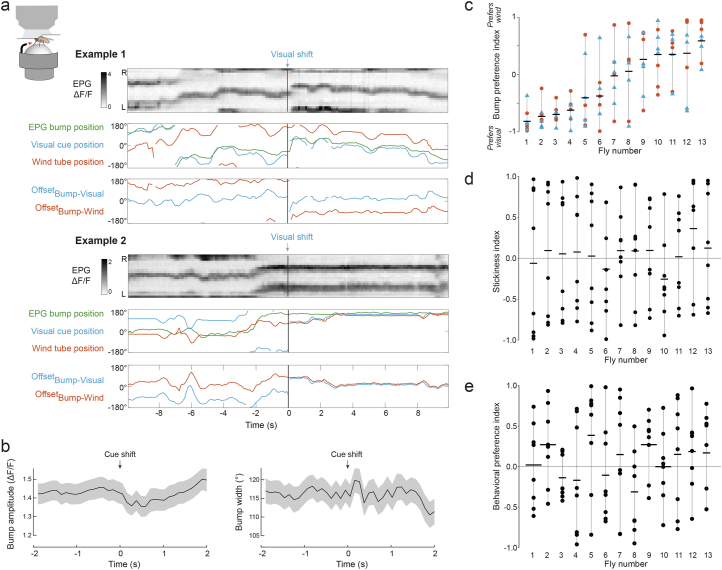
Effects of cue shifts. This figure shows additional details regarding the 13 flies in Fig. 4. **a**. Responses to cue shifts. In both these examples, the visual cue shifted while the wind did not shift. In Example 1, the EPG bump follows the shift in the visual cue, so that the offset relative to that cue is restored. In Example 2, when the visual cue shifts, the bump does not shift, and so it retains its offset relative to the wind. **b**. Mean bump amplitude and mean bump width (± s.e.m.) around the time of cue shifts. Cue shifts produce no significant change in bump amplitude or width (p = 0.10 for bump amplitude, p = 0.26 for bump width, 2-sided Wilcoxon signed rank tests, comparing 1 sec before vs 1 sec after a shift). **c**. Bump preference index for each cue shift, for each fly, with flies sorted by mean bump preference index. Each thin vertical line connects the data for 4 wind shifts (orange) and 4 visual shifts (blue) for the same fly. Thick horizontal lines show mean values for each fly. This plot reproduces data from Fig. 4e. **d**. Same but for the “stickiness index”. This index is a metric for how much the bump tends to follow the cue that does not shift (that is, how sticky’ the bump is). A value of +1 means that the bump sticks with the non-shifted cue, whereas a value of -1 means that the bump follows the shifted cue. There were no flies where the bump tended to prefer either the shifted cue or the non-shifted cue. Flies are sorted as in (**c**). **e**. Same but for the “behavioral preference index”. Here, -1 means the visual cue dominates the fly’s behavioral orientation, whereas +1 means the wind dominates. For example, if the wind shifts by -120° and the fly then executes a -120° turn, the index would be +1 for that trial. The mean behavioral preference index was near zero for all individuals, meaning they reoriented with equal frequency in response to visual cue shifts and wind shifts. This was true even in the flies with a strongly biased HD system (for example, Fly 1 or Fly 13).

**Extended Data Fig. 4:**
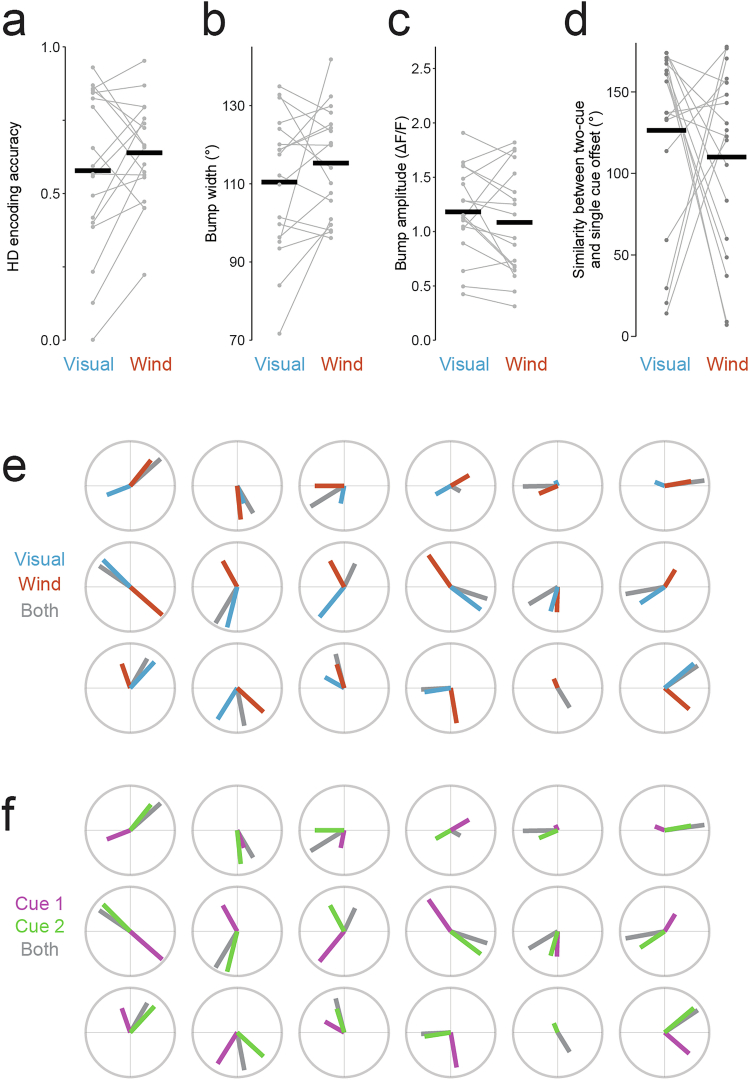
Comparing one-cue environments and a two-cue environment. This figure shows additional detail for the 18 flies in Fig. 5. **a**–**c**. There is no difference between the visual cue and the wind in terms of HD encoding accuracy (p = 0.28; **a**), bump width (p = 0.27; **b**), or bump amplitude (p = 0.20, paired 2-sided Wilcoxon tests; **c**). These plots show data from the two initial single-cue environments, prior to the onset of the two-cue environment. Thick horizontal lines are mean values. **d**. The offset during the two-cue block is equally similar to the initial offset with respect to the visual cue and the initial offset with respect to the wind (p = 0.52, paired Wilcoxon test). **e**. Offset data for both initial single-cue environments and the two-cue environment, for all 18 flies. The angle of each line indicates the mean offset during that block; the length of each line indicates the vector strength of the offset (that is, HD encoding accuracy). Note that there is no relationship between the initial visual offset and the initial wind offset. Note also that there is no tendency for the visual cue or the wind to dominate the offset in the two-cue period. **f**. Same but now color-coded by cue order. Note that the offset in the two-cue environment is generally similar to the offset in the environment with cue 2. The exceptions to this rule are generally flies where the offset was not stable (for example, the fourth fly in the top row, or the fifth fly in the bottom row).

**Extended Data Fig. 5:**
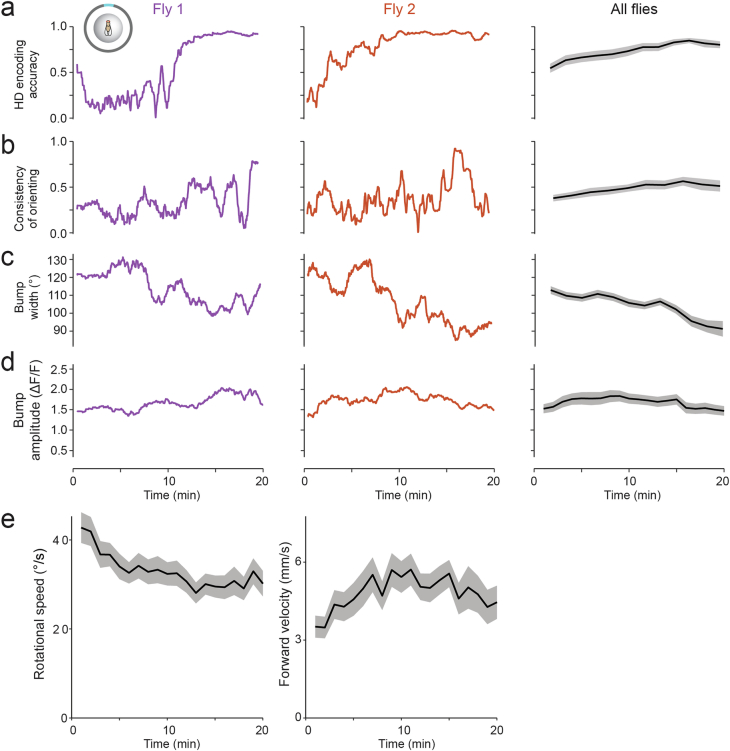
Acquiring familiarity with a virtual environment. This figure shows data from 28 flies walking in the environment with the bright visual cue, beginning when they are first exposed to this environment. In **a-c**, The first two plots show data from two example flies and the third plot shows data for all flies (mean ± s.e.m). Each variable was smoothed with a 60-s moving window, after first excluding time points where the fly was immobile or goodness of fit was below threshold. **a**. Mean HD encoding accuracy increases over time. Over all flies, there is a significant effect of time in a linear mixed-effects model (p < 0.001). **b**. Mean consistency of behavioral orientation increases over time. Over all flies, there is a significant effect of time in a linear mixed-effects model (p < 0.001). **c**. Mean bump width decreases over time. Over all flies, there is a significant effect of time in a linear mixed-effects model (p < 0.001). **d**. Mean bump amplitude increases during the first 10 min. in the environment, and then decreases. Over all flies, there is a significant effect of time in a linear mixed-effects model (p = 0.03). **e**. Mean rotational speed and forward velocity for the same flies over this time period (± s.e.m).

In Fig. 4 and Extended Data Fig. 3, we computed a bump preference index for each cue shift. Here, we first obtained the mean value of the visual cue and wind offset before the cue shift (calculated over the 2-min window preceding each shift) and following the cue shift (calculated over a 2-min window starting 30 s after the cue shift because we found that it took about 30 s for the offset to stabilize after a cue shift). We then calculated the change in visual cue offset and wind offset by taking the difference between the postshift and preshift values. We computed the bump preference index as follows:

$$\begin{array}{l}{\rm{bump}}\,{\rm{preference}}\,{\rm{index}}=\frac{|{\rm{change}}\,{\rm{in}}\,{\rm{visual}}\,{\rm{cue}}\,{\rm{offset}}|-|{\rm{change}}\,{\rm{in}}\,{\rm{wind}}\,{\rm{offset}}|}{|{\rm{change}}\,{\rm{in}}\,{\rm{visual}}\,{\rm{cue}}\,{\rm{offset}}|+|{\rm{change}}\,{\rm{in}}\,{\rm{wind}}\,{\rm{offset}}|}\end{array}$$

The stickiness index was obtained in the same way, except that the offset here was computed relative to the shifted cue and the nonshifted cue. Here, a value of +1 means that the bump ‘sticks’ with the nonshifted cue, whereas a value of −1 means that the bump follows the shifted cue:

$$\begin{array}{l}{\rm{stickiness}}\,{\rm{index}}=\frac{|{\rm{change}}\,{\rm{in}}\,{\rm{shifted}}_{-}{\rm{cue}}\,{\rm{offset}}|-|{\rm{change}}\,{\rm{in}}\,{\rm{non}}{\rm{shifted}}_{-}{\rm{cue}}\,{\rm{offset}}|}{|{\rm{change}}\,{\rm{in}}\,{\rm{shifted}}_{-}{\rm{cue}}\,{\rm{offset}}|+|{\rm{change}}\,{\rm{in}}\,{\rm{non}}{\rm{shifted}}_{-}{\rm{cue}}\,{\rm{offset}}|}\end{array}$$

The behavioral preference index was computed in the same way, except that the offset here was the angular difference between the fly’s HD and the cue position:

$$\begin{array}{l}{\rm{behavioral}}\,{\rm{preference}}\,{\rm{index}}=\frac{|{\rm{change}}\,{\rm{in}}\,{\rm{visual}}\,{\rm{cue}}\,{\rm{offset}}|-|{\rm{change}}\,{\rm{in}}\,{\rm{wind}}\,{\rm{offset}}|}{|{\rm{change}}\,{\rm{in}}\,{\rm{visual}}\,{\rm{cue}}\,{\rm{offset}}|+|{\rm{change}}\,{\rm{in}}\,{\rm{wind}}\,{\rm{offset}}|}\end{array}$$

#### Offset changes during configurational learning

In Fig. 5, we defined the amount of conflict in the two-cue environment as follows:

$${\theta }_{n}={\rm{offset}}\; {\rm{with}}\; {\rm{both}}\; {\rm{cues}}-{\rm{initial}}\; {\rm{single}}\; {\rm{cue}}\; {\rm{offset}}$$

The resulting offset change was determined as follows:

$${\phi }_{n}={\rm{final}}\; {\rm{single}}\; {\rm{cue}}\; {\rm{offset}}-{\rm{initial}}\; {\rm{single}}\; {\rm{cue}}\; {\rm{offset}}$$

In Fig. 5d, to fit the relationship between the conflict *θ*_*n*_ and the amount of remapping ${\phi }_{n}$, we used a probabilistic model:

$${\mu }_{n}=a{\cdot \theta }_{n}+b$$

$${\phi }_{n}\sim {\rm{von}}\; {\rm{Mises}}(\,{\mu }_{n},\kappa )$$

In other words, we assumed that ${\phi }_{n}$ is generated by adding a noise (distributed according to the von Mises distribution) to an intermediate variable ${\mu }_{n}$, which is assumed to be a linear function of ${\theta }_{n}$ with slope *a* and offset *b*. The magnitude of the noise is characterized by the concentration parameter *κ* of the von Mises distribution. We obtained estimates of parameters *α*, *b* and *κ* by performing Bayesian analysis with the following prior distributions over the parameters:

$$a\sim N(0,1)$$

$$b\sim {\rm{uniform}}(-\uppi ,\uppi )$$

$$\kappa \sim {\rm{inverse}}\; {\rm{gamma}}(1.91,6.47)$$

We chose a prior over parameter *a* on the basis of prior knowledge that a slope with large magnitude is highly unlikely. We chose a uniform prior over the offset *b*. We chose an inverse gamma distribution over the concentration parameter *κ* to suppress large *κ* values (all points lie perfectly on a straight line) and to strongly suppress small *κ* values (large noise)^68^. The parameters of the inverse gamma were chosen such that <2% of *κ* fell below 1 or above 50. We confirmed that these prior choices are reasonable according to prior predictive simulations.

We estimated the three parameters by obtaining samples from the posterior using the Hamiltonian Monte Carlo implemented in Stan 2.19.0 (ref. ^66^) (four chains with 1,000 samples each after 1,000 samples of warm-up, no thinning) accessed using PyStan2. Diagnostics of the Hamiltonian Monte Carlo did not show any issues with the fit, examination of the posterior distribution using the pair plots indicated no signs of multimodality and the posterior retrodictive checks showed no obvious discrepancies between the model and the data^69^.

#### Remapping in inverted gain

**Extended Data Fig. 6:**
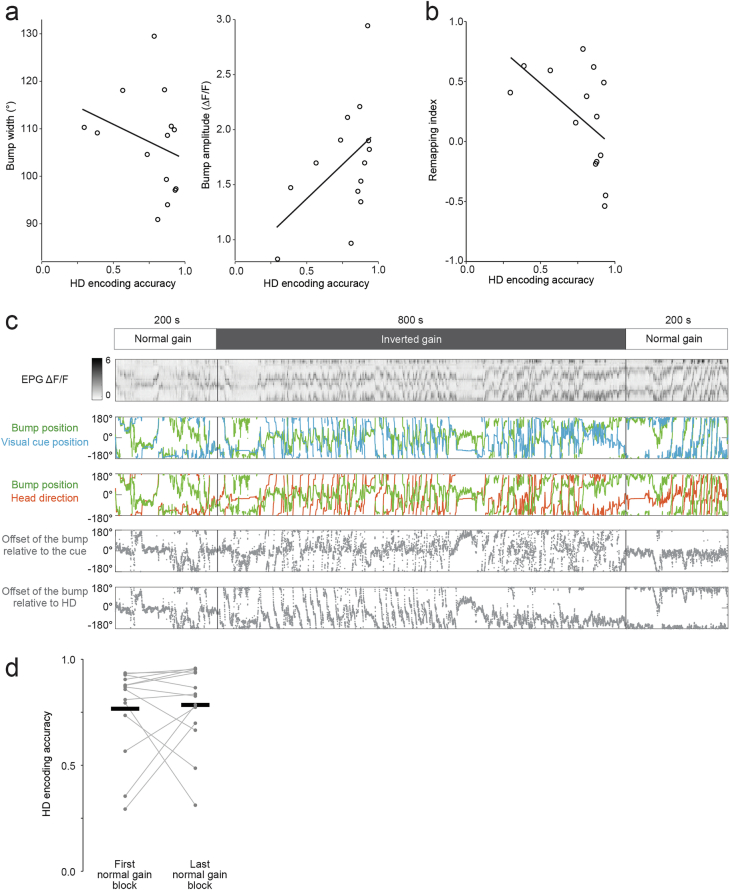
Learning in inverted gain. This figure shows additional details regarding the 14 flies in Fig. 7. **a**. In the initial normal-gain segment, bump width is significantly negatively correlated with HD encoding accuracy (p < 0.001, Pearson’s correlation). There is a trend for bump amplitude to be positively correlated with HD encoding accuracy, but this relationship falls short of statistical significance in this group of flies (p = 0.08, Pearson’s correlation). **b**. There is a trend for the remapping index (in the final part of the inverted gain block) to be negatively correlated with HD encoding accuracy (in the preceding normal gain block), but this relationship falls short of statistical significance (p = 0.06). **c**. We finished each experiment by returning to normal gain for 200 s. This panel shows the entire experiment (including this last normal gain block) for one example fly. This is the same example fly shown in Fig. 7b. Note that the EPG bump tracks HD accurately during the final normal gain block. **d**. On average, HD encoding accuracy was no different in the first and last normal gain block (p = 0.67, paired 2-sided Wilcoxon test).

**Extended Data Fig. 7:**
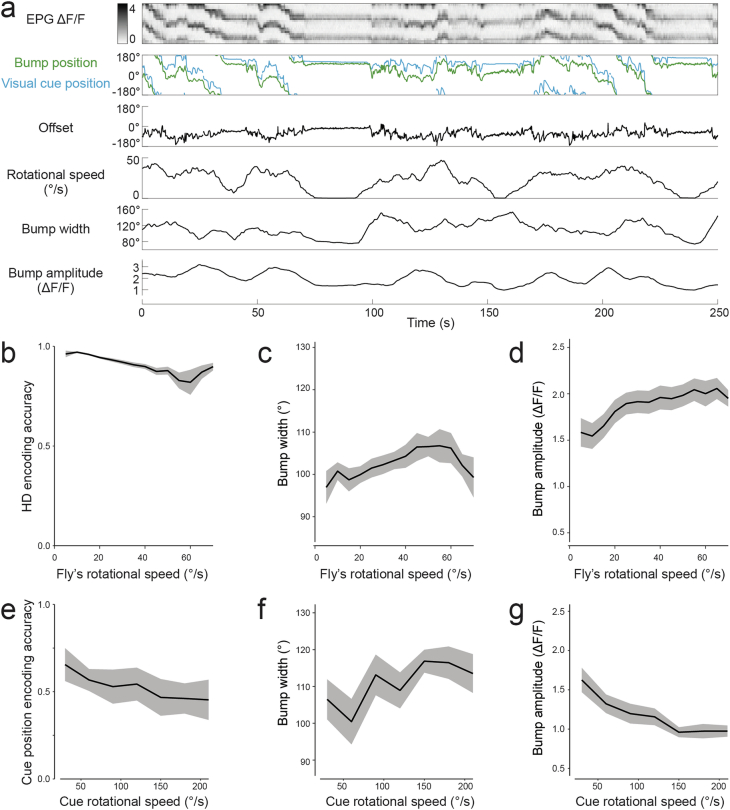
Correlations between bump profile and rotational speed. Panels **a-d** show data from 28 flies walking in a virtual reality environment with a bright visual cue. Each variable was smoothed with a 10-s moving window, after first excluding time points where the fly was immobile or goodness of fit was below threshold. **a**. Example fly. Here and elsewhere in this figure, we used a 10-s rolling window to compute the rotational speed, bump width and bump amplitude values. **b**. Mean HD encoding accuracy (± s.e.m.) versus the fly’s rotational speed. As the fly’s rotational speed increases, HD encoding accuracy decreases (linear mixed-effects model, p < 0.001). **c**. Mean z-scored bump width (± s.e.m.) versus the fly’s rotational speed. As the fly’s rotational speed increases, so does bump width (linear mixed-effects model, p < 0.001). **d**. Mean z-scored bump amplitude (± s.e.m.) versus the fly’s rotational speed. As the fly’s rotational speed increases, so does the bump amplitude (linear mixed-effects model, p < 0.001). Panels e-g show data from 7 flies tested in separate experiments where we displayed a bright visual cue rotating around the fly. The cue rotated at a different speed for each block of the experiment. **e**. Mean cue position encoding accuracy (± s.e.m.) versus cue rotational speed. Cue position encoding accuracy is computed in the same way that we compute HD encoding accuracy, except that it measures encoding of cue position rather than HD. For example, a value of 1 would mean that the EPG bump perfectly tracks the cue, whereas a value of 0 would mean that the bump moves independently from the cue. Cue position encoding accuracy decreases as cue speed increases (linear mixed-effects model, p < 0.001). **f**. Mean bump width (± s.e.m.) versus cue rotational speed. Bump width increases as cue speed increases (linear mixed-effects model, p = 0.007). **g**. Mean bump amplitude (± s.e.m.) versus cue rotational speed. Bump amplitude decreases as cue speed increases (linear mixed-effects model, p < 0.001).

**Extended Data Fig. 8:**
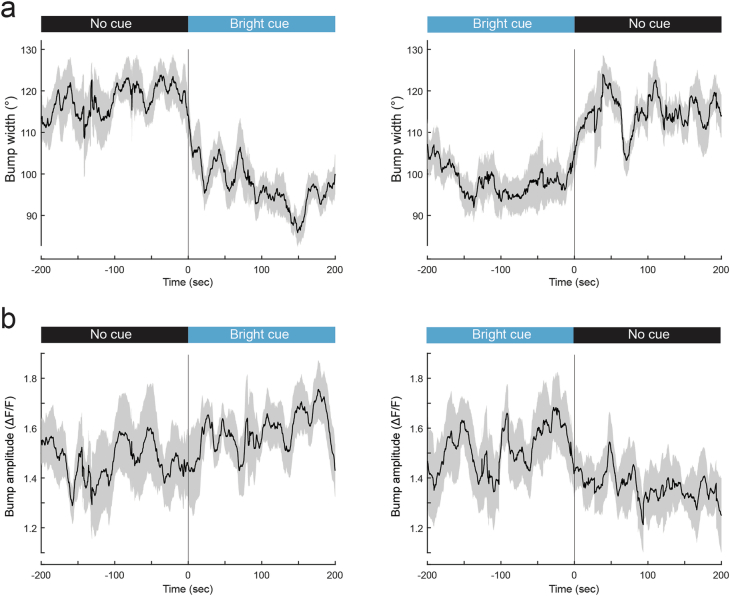
Bump profile around the time of a cue change. **a**. Bump width over time (mean ± s.e.m.), around transitions between darkness and the bright visual cue. This plot shows data from the 15 flies in Fig. 2. Note that bump width decreases abruptly at the time of cue onset, and it increases abruptly at the time of cue offset. **b**. Same but for bump amplitude.

In Fig. 7 and Extended Data Fig. 6, the remapping index is the difference between the HD encoding accuracy with respect to the fly’s HD and the HD encoding accuracy with respect to the visual cue position, excluding time points where the fly was immobile. Specifically,

$$\begin{array}{l}{\rm{remapping}}\; {\rm{index}}\\={\rm{vector}}\;{\rm{strength}}\left({\rm{offset}}_{{\rm{bump}}-{\rm{HD}}}\right)-{\rm{vector}}\;{\rm{strength}}({\rm{offset}}_{{\rm{bump}}-{\rm{visual}}})\end{array}$$

Data points when the fly was immobile were discarded from the remapping index calculation. This index is designed to be robust to the spontaneous alterations in the fly’s behavior. For example, if the fly were to walk perfectly straight for some time and if its EPG activity bump did not move during this time, then vector strength(offset_bump−HD_) and vector strength(offset_bump−visual_) would both be 1; taking the difference between these two vector strength values yields a remapping index of 0, which captures the fact that the bump would be equally well aligned with the fly’s self-motion and the visual cue.

### Network model

Our network model follows the structure of a previously published ring attractor model^22,24^, with several key modifications. First, we systematically varied the amplitude of sensory (ER) inputs to the ring attractor. Second, we gave the attractor network two independent streams of sensory input, with distinct ER → EPG weights. Third, we updated two learning parameters (*w*_max_ and *g*_0_) to promote stable outcomes after learning, while also maintaining the sensitivity of the network to all available cues. Details on these changes are provided below, along with a summary of the model infrastructure.

In this model, the firing rate dynamics of the *N* simulated EPG neurons are given by

$$\begin{array}{l}\tau \frac{d{f}_{n}}{dt}=-{f}_{n}+\left[\alpha {f}_{n}+D(\;{f}_{n-1}+{f}_{n+1})\right.\\\qquad\quad\;\left.+\frac{v(t)}{{v}_{rel}}\frac{1}{2}(\;{f}_{n+1}-{f}_{n})-\beta \sum _{l}{f}_{l}+{I}_{n,1}(t)+{I}_{n,2}(t)+1\right]_{+}\end{array}$$

where *f*_*n*_ is the firing rate of neuron *n*, $\tau$ is the network’s time constant, ${\left[\cdot \right]}_{+}$ is the linear-rectifying function that returns ${\left[a\right]}_{+}=a$ if $a > 0$ and ${\left[a\right]}_{+}=0$ otherwise, *α* and *D* control the local self-excitation, $v(t)$ is the fly’s current angular velocity, ${v}_{\rm{rel}}$ is a parameter that controls how this angular velocity impacts EPG activity, *β* controls the amount of global inhibition and ${I}_{n,k}(t)$ is the external input from cue $k\in \{\mathrm{1,2}\}$ to EPG neuron *n*.

Inhibitory inputs indicating cue positions, ${I}_{k}={({I}_{1,k},\cdots ,{I}_{N,k})}^{T}$, are formed by

$${I}_{k}(t)=-{W}_{k}(t){g}_{k}({t})$$

where *g*_*k*_ = (*g*_1,*k*,…,*M*,*k*_) is the vector defining the activity of the *M* ER neurons associated with cue *k* and *W*_*k*_ is the *N* × *M* weight matrix whose *nm*th element *W*_*k*,*nm*_ specifies the synaptic weight from the *m*th ER neuron to the *n*th EPG neuron. Weights are non-negative; thus, *I*_*k*_ ≤ 0. These synaptic weights are continuously updated according to a postsynaptically gated learning rule

$$\frac{{\mathrm{d}}{W}_{k,nm}}{{\mathrm{d}}t}=\eta |v(t)|{f}_{n}({w}_{{\max }}(1-{g}_{k,m}(t)/{g}_{0})-{W}_{k,nm}(t))$$

where *η*(|*v*(*t*)|) is the learning rate (which depends on the absolute value of the fly’s rotational velocity *v*(*t*)) and *w*_max_ and *g*_0_ are parameters that control the learning dynamics of the different parts of the learning rule. This learning rule can be rewritten as follows:

$$\frac{d{W}_{k,nm}}{dt}=\eta |v(t)|{f}_{n}({w}_{{\max }}-{W}_{k,nm}(t))-\eta (|v(t)|){f}_{n}{w}_{{\max }}{g}_{k,m}(t)/{g}_{0}$$

where the first term represents nonassociative LTP and the second term represents associative LTD. Here, nonassociative LTP depends on postsynaptic activity but not presynaptic activity; by contrast, associative LTD depends on both presynaptic and postsynaptic activity. Activity of the *k*th ER neuron population is given by

$${g}_{k,m}(t)={\varepsilon }_{\rm{ER}}(t)+{A}_{k}\exp [{\kappa }_{k}\cos (\theta (t)-{\theta }_{m})-1]$$

where the upper bound of the uniformly distributed baseline noise ${\varepsilon }_{\rm{ER}}(t)\sim {\rm{uniform}}(0,{b}_{\rm{ER}}{\sum }_{l}\;{{f}_{l}}^{\rm{ss}})$ is the summed activity of the EPG neurons in a steady state without external inputs, ${I}_{n,1}(t)={I}_{n,2}(t)=0$, scaled by a constant factor *b*_ER_, *A*_*k*_ is the amplitude of the population activity profile (ER amplitude), *κ*_*k*_ is the precision (inverse width) of the population activity profile and *θ*_*m*_ is the preferred HD of the *m*th ER neuron.

We simulated the fly’s HD sequence by drawing a sequence of HD displacements, ${{\mathrm{d}}u}(t)\sim{N}({0},{{\sigma }_{u}}^{2}{{\mathrm{d}}t})$, from a zero-mean Gaussian with variance ${{\sigma }_{u}}^{2}{{\mathrm{d}}t}$, which we then turned into a sequence of angular velocities ${{\mathrm{d}}u}(t)/{{\mathrm{d}}t}$. To ensure a smooth angular velocity sequence, we then applied a running window average of the ${{\mathrm{d}}u}(t)/{{\mathrm{d}}t}$ sequence (centered window, size 2.5 s) to generate the actual angular velocity sequence ${v}_{\rm{true}}(t)$. This sequence was integrated across time to yield the HD sequence ${\theta }_{\rm{true}}(t)$. The activity of the EPG neurons *f*_*n*_ was driven by a noisy version of this angular velocity, $v(t)={v}_{\rm{true}}(t)+{\varepsilon }_{\rm{AV}}(t)$, where ${\varepsilon }_{\rm{AV}}(t)$ is a Gaussian white noise process with s.d. ${\sigma }_{\rm{AV}}$ that has been smoothed with a running window average (centered window, size 0.04 s).

In all network simulations, we either simulated individual trials or simulated a set of trials and then averaged across those trials. In each trial, the EPG activity and synaptic weight dynamics were simulated by Euler integration and the ER activity and the fly’s HD sequence were generated in time steps of *Δt* = 2.5 ms. The EPG activity was initialized by a cosine profile and then simulating the network for 20 s in the absence of both angular velocity and external inputs, ${I}_{n,1}(t)={I}_{n,2}(t)=0$, such that the EPG activity reached an approximate steady state *f*^ss^. The synaptic weights were initialized randomly by drawing their elements from a uniform distribution over [${{0},{1}}$) and subsequently normalized to reach an initial matrix Frobenius norm of ${||}{W}_{k}|{|}_{F}=1.5$. This was followed by a burn-in period (analysis-dependent duration; discussed below) such that synaptic weights could approximately reach a steady state. What followed was dependent on the specific analysis in question and is described in detail below. Unless otherwise mentioned, we simulated a network with $N=M=32$ EPG and ER neurons (per external input) and used the following parameters: EPG network time constant $\tau =50$ ms, local excitation $\alpha =-8.93$ and $D=5.19$, global inhibition $\beta =0.11$, angular velocity scaling ${v}_{\rm{rel}}=3.64$, synaptic weight learning rate $\eta =0.34$, learning parameters ${w}_{\max}=1/17$ and ${g}_{0}=1$, input activity baseline noise factor ${b}_{\rm{ER}}=0.45$, fly motion noise ${\sigma }_{u}=8$ radians per second and angular velocity noise ${\sigma }_{\rm{AV}}=1$ radians per second. Note that these values of *w*_max_ and *g*_0_ are different from those used in previous studies; we found that it was necessary to change these values to balance stability and flexibility during learning. In particular, if learning led to weight magnitudes that were too large, the network model ignored angular velocity inputs; on the other hand, if learning led to weight magnitudes that were too small, the network model never established a stable ER → EPG mapping. To simulate the ER activity ${g}_{k}(t)$, we specified a scale factor $\widetilde{{A}_{k}}$ (analysis-dependent scale factor; discussed below) that was multiplied by the amplitude of the steady-state EPG activity bump *f*^ss^ (~1.062) to obtain the ER amplitude ${A}_{k}\approx 1.062\widetilde{{A}_{k}}$. For all simulations, we set the width of the ER activity bump to be ${w}_{k}=0.8$ for all *k*, which was then converted to the precision by ${\kappa }_{k}=\log (2)/(1-{\mathrm{cos}} ({w}_{k}/2))$. For simulations in which only a single cue was present, we fixed ${I}_{2,n}=0$ for all *n*. In the list of parameter values above, all reported times are simulation times. When plotting time courses, we converted them to experiment time by assuming that 24 s of experiment time corresponded to 1 s of simulation time. We assessed HD encoding accuracy by first computing the circular variance between HD *θ*_true_ and the position of the EPG activity bump ${\mathrm{arg}}\mathop{\mathrm{max }}\limits_{l}{f}_{l}$ on $[-{\uppi} ,\,{\uppi} )$ over a causal 8-s window. The HD encoding accuracy was defined as $1-{\rm{circular}}\; {\rm{variance}}$. We computed bump width and amplitude by finding the full width at half maximum and the difference between the peak and the trough, respectively, of the EPG activity bump. We computed the weight matrix notch depth by first smoothing the weight matrix *W*_*k*_ with a Gaussian filter with s.d. ${\sigma }_{\rm{smooth}}=2$ and then finding the difference between the maximum and the minimum matrix elements.

#### Impact of cue intensity on bump parameters and across-individual variability

In Figs. 2h,i and 3d,f, we simulated a network with a single external input with different cue intensities (41 values of $\widetilde{{A}_{k}}$ from 0 to 2 in steps of 0.05). We assessed the bump parameters in a 30-s period after a burn-in period of 120 s. The bump parameters shown in Fig. 2h are averages across 100 simulated trials for each cue intensity. To simulate across-individual variability, we generated individual variations in ER amplitude in response to a given cue. We, thus, plotted the bump width (Fig. 3d) and bump amplitude (Fig. 3f) over HD encoding accuracy for different cue intensity (11 values of $\widetilde{{A}_{k}}$ from 0.5 to 1 in steps of 0.05) and five different simulated trials per cue intensity.

#### Impact of individual variations in experienced cue intensity on cue weighting

In Fig. 4f,g, we simulated a network with two external inputs. To introduce variations and differences in experienced cue intensity, we varied the ER amplitude (11 values of $\widetilde{{A}_{k}}$ from 0 to 1 in steps of 0.05) of one ER population while holding the ER amplitude of the other ER population constant (ER amplitude scale set to 0.75) and vice versa, producing 22 ER amplitude pairs. ER amplitude was set to 0 when the associated cue was off. Each simulated trial began with a 30-s burn-in period during which both cues were off. The burn-in period was followed by two single-cue blocks where each cue was turned on for 25 s one at a time. We assessed the bump width, amplitude and HD encoding accuracy during the second half of each single-cue block. After the single-cue blocks, both cues were on for a total of 262.5 s. Every 12.5 s, one of the cues shifted, with the shifts alternating between visual cue and wind cue. Thus, each simulated trial comprised 20 cue shifts with ten shifts per modality. Cue shifts were +120° or −120°, with the direction determined randomly for each shift. If visual cue was on in the first single-cue block, then visual cue was the first cue to shift and vice versa. The order was balanced across trials such that visual cue shifted first in half of the trials. For each cue shift, we computed a bump preference index (discussed above) using simulated data from a 5-s period before and a 5-s period after the cue shift. We simulated three trials for each combination of ER amplitude pair and cue order, producing 132 trials in total. The scatter points in Fig. 4f and vertical lines in Fig. 4g each represent a single simulated trial. Each scatter point in Fig. 4g is an average across ten cue shifts of the same modality from the same trial.

#### Temporal evolution of bump parameters during cue combination

In Fig. 5f,g, we simulated a network with two equally strong external inputs. When a cue was on, its associated $\widetilde{{A}_{k}}$ was set to 2; when a cue was off, its associated $\widetilde{{A}_{k}}$ was set to 0. Each simulated trial began with a burn-in period of 30 s during which both cues were off. The burn-in period was followed by two 25-s single-cue blocks; cue 1 was on while cue 2 was off in the first single-cue block and vice versa in the second single-cue block. This was followed by a two-cue block during which both cues were on for 32.5 s. The two-cue block was followed by two 12.5-s single-cue blocks, again with cue 1 on in the first and cue 2 on in the second. We visualized the temporal evolution of the bump parameters after the burn-in period (Fig. 5f). The bump parameters shown in Fig. 5f are averages across 100 simulated trials.

#### Impact of variation in ER amplitude on remapping during inverted gain

In Fig. 7g,h, to model across-individual variability, we simulated a network with a single external input with five values of $\widetilde{{A}_{k}}$ ranging from 1 to 2 in steps of 0.25. Each simulated trial began with a burn-in period of 60 s during which the gain between the simulated fly’s movement and the cue movement was set to 1. Following the burn-in period, the gain remained at 1 for another 8 s (normal gain), after which it was set to −1 for 32 s (inverted gain). We visualized the temporal evolution of the bump parameters after the burn-in period (Fig. 7g). The bump parameters shown in Fig. 7g are averages across 100 simulated trials for each ER amplitude.

### Reporting summary

Further information on research design is available in the Nature Portfolio Reporting Summary linked to this article.

## Online content

Any methods, additional references, Nature Portfolio reporting summaries, source data, extended data, supplementary information, acknowledgements, peer review information; details of author contributions and competing interests; and statements of data and code availability are available at 10.1038/s41593-024-01823-z.

## Supplementary information

Reporting Summary

## Data Availability

All data are available from the DANDI repository (https://dandiarchive.org/dandiset/000289).

## Code availability

Data analysis code is available via Zenodo at 10.5281/zenodo.15733533 (ref. ^70^). The model code is also available via Zenodo at 10.5281/zenodo.13270653 (ref. ^71^). Simulated data to recreate figures with the model code are available via figshare at 10.6084/m9.figshare.26510239 (ref. ^72^).
