# Supplementary material for: Multimodal cue integration and learning in a neural representation of head direction
Source: Nat Neurosci. 2025 Jul 23;28(8):1729–40. doi: 10.1038/s41593-024-01823-z (PMC12321575; doi:10.1038/s41593-024-01823-z)
Supplement: Supplementary file 1 — Reporting Summary [file 41593_2024_1823_MOESM1_ESM.pdf]

nature portfolio

Corresponding author(s): Rachel I. Wilson

Last updated by author(s): 2024/07/30

Reporting Summary

Nature Portfolio wishes to improve the reproducibility of the work that we publish. This form provides structure for consistency and transparency in reporting. For further information on Nature Portfolio policies, see our Editorial Policies and the Editorial Policy Checklist.

Statistics

For all statistical analyses, confirm that the following items are present in the figure legend, table legend, main text, or Methods section.

n/a	Confirmed	
☐	☒	The exact sample size (*n*) for each experimental group/condition, given as a discrete number and unit of measurement
☐	☒	A statement on whether measurements were taken from distinct samples or whether the same sample was measured repeatedly
☐	☒	The statistical test(s) used AND whether they are one- or two-sided<br>*Only common tests should be described solely by name; describe more complex techniques in the Methods section.*
☐	☒	A description of all covariates tested
☐	☒	A description of any assumptions or corrections, such as tests of normality and adjustment for multiple comparisons
☐	☒	A full description of the statistical parameters including central tendency (e.g. means) or other basic estimates (e.g. regression coefficient) AND variation (e.g. standard deviation) or associated estimates of uncertainty (e.g. confidence intervals)
☒	☐	For null hypothesis testing, the test statistic (e.g. *F*, *t*, *r*) with confidence intervals, effect sizes, degrees of freedom and *P* value noted<br>*Give P values as exact values whenever suitable.*
☐	☒	For Bayesian analysis, information on the choice of priors and Markov chain Monte Carlo settings
☒	☐	For hierarchical and complex designs, identification of the appropriate level for tests and full reporting of outcomes
☒	☐	Estimates of effect sizes (e.g. Cohen's *d*, Pearson's *r*), indicating how they were calculated

Our web collection on statistics for biologists contains articles on many of the points above.

Software and code

Policy information about availability of computer code

Data collection	Matlab 2020a, 2021a; ScanImage 2018b or 2020; FicTrac v2.1.1; Python 3.7, 3.9
Data analysis	Matlab 2020a, 2021a; Python 3.7, 3.9; RStudio 2022.02.0; R 4.1.3; Stan 2.19.0; PyStan 2.19.0; NoRMCorre

For manuscripts utilizing custom algorithms or software that are central to the research but not yet described in published literature, software must be made available to editors and reviewers. We strongly encourage code deposition in a community repository (e.g. GitHub). See the Nature Portfolio guidelines for submitting code & software for further information.

Data

Policy information about availability of data

All manuscripts must include a data availability statement. This statement should provide the following information, where applicable:

- Accession codes, unique identifiers, or web links for publicly available datasets
- A description of any restrictions on data availability
- For clinical datasets or third party data, please ensure that the statement adheres to our policy

All data are available from the DANDI repository (https://dandiarchive.org/dandiset/000289).

Human research participants

Policy information about studies involving human research participants and Sex and Gender in Research.

Reporting on sex and gender	n/a
Population characteristics	n/a
Recruitment	n/a
Ethics oversight	n/a

Note that full information on the approval of the study protocol must also be provided in the manuscript.

Field-specific reporting

Please select the one below that is the best fit for your research. If you are not sure, read the appropriate sections before making your selection.

☒ Life sciences ☐ Behavioural & social sciences ☐ Ecological, evolutionary & environmental sciences

For a reference copy of the document with all sections, see nature.com/documents/nr-reporting-summary-flat.pdf

Life sciences study design

All studies must disclose on these points even when the disclosure is negative.

Sample size	All sample sizes were chosen based on standard sample sizes in the field. These sample sizes are conventionally determined on the basis of the expected magnitude of animal-to-animal variability, given published results and pilot data. Our sample sizes are similar to those reported in previous publications (refs 23,33,34,37,38,62).
Data exclusions	No flies were excluded from analyses.
Replication	All results reported are based on multiple replicates, with n values stated in the legends. All of the results in the study were found to be reproducible.
Randomization	Following the initial period with a bright cue, the order of the different brightness blocks in the experiment displayed in Figures 2-3 and Extended Data Figures 2 and 8 was randomized, as noted in the Methods.<br>The experimental configuration assigned to each fly for the experiment corresponding to figs. 4-5 and sup figs 3-4 (order of wind and visual cues) was also randomized.
Blinding	The experimenter was not blind to the experimental conditions assigned to each fly. Blinding is only potentially relevant to experiments where different flies experienced different experimental treatments (e.g., different genotypes or different drug treatments). However, the was only one experiment in the paper where different flies experienced different experimental treatments, namely the cases where the order of the different brightness blocks was randomized (Figures 2-3 and Extended Data Figures 2 and 8). Here, the experimenter could not be blind to the experimental condition because the stimulus apparatus delivered the stimulus in a manner that was visible to the experimenter.

Reporting for specific materials, systems and methods

We require information from authors about some types of materials, experimental systems and methods used in many studies. Here, indicate whether each material, system or method listed is relevant to your study. If you are not sure if a list item applies to your research, read the appropriate section before selecting a response.

Materials & experimental systems

n/a	Involved in the study	
☒	☐	Antibodies
☒	☐	Eukaryotic cell lines
☒	☐	Palaeontology and archaeology
☐	☒	Animals and other organisms
☒	☐	Clinical data
☒	☐	Dual use research of concern

Methods

n/a	Involved in the study	
☒	☐	ChIP-seq
☒	☐	Flow cytometry
☒	☐	MRI-based neuroimaging

Animals and other research organisms

Policy information about studies involving animals; ARRIVE guidelines recommended for reporting animal research, and Sex and Gender in Research

Laboratory animals	All experimental animals were 1 day old virgin Drosophila melanogaster with the genotype w/+ ; + ; P{R60D05-Gal4}attP2 / P{20XUAS-IVS-jGCaMP7f}VK00005. Both P{R60D05-Gal4}attP2 and P{20XUAS-IVS-jGCaMP7f}VK00005 were obtained from the Bloomington Drosophila Stock Center (RRID:BDSC_39247 and RRID:BDSC_79031, respectively). Virgin female flies were anesthetized on CO2 and collected at least 12 h before the experiment, then allowed to recover on molasses food. Imaging experiments were conducted the following day, 14-36 h after eclosion.
Wild animals	No wild animals were used in this study.
Reporting on sex	Given their larger size, which makes dissections easier, and usually results in better behavior in tethered animals, we relied purely on females for this study. This is a standard practice for researchers in the field conducting neurophysiological experiments in fruit flies.
Field-collected samples	No field samples were collected for this study.
Ethics oversight	No ethical approval was required because experiments were performed on Drosophila melanogaster.

Note that full information on the approval of the study protocol must also be provided in the manuscript.
